# Synthesis of new diphenyl urea-clubbed imine analogs and its Implications in diabetic management through in vitro and in silico approaches

**DOI:** 10.1038/s41598-023-28828-1

**Published:** 2023-02-01

**Authors:** Anam Rubbab Pasha, Ajmal Khan, Saeed Ullah, Sobia Ahsan Halim, Javid Hussain, Muhammad Khalid, Muhammad Moazzam Naseer, Attalla F. El-kott, Sally Negm, Ahmed Al-Harrasi, Zahid Shafiq

**Affiliations:** 1 Institute of Chemical Sciences, Bahauddin Zakariya University, Multan, 60800 Pakistan; 2 Natural and Medical Sciences Research Center, University of Nizwa, Birkat-ul-Mouz 616, Nizwa, Sultanate of Oman; 3 International Center for Chemical and Biological Sciences, H. E. J. Research Institute of Chemistry, University of Karachi, Karachi, 75270 Pakistan; 4 Department of Chemistry, Khwaja Fareed University of Engineering and Information Technology, Rahim Yar Khan, 64200 Pakistan; 5 Centre for Theoretical and Computational Research, Khwaja Fareed University of Engineering and Information Technology, Rahim Yar Khan, 64200 Pakistan; 6 Department of Chemistry, Quaid-i- Azam University, Islamabad, 45320 Pakistan; 7 Department of Biology, College of Science, King Khalid University, 61421 Abha, Saudi Arabia; 8 Department of Zoology, College of Science, Damanhour University, Damanhour, 22511 Egypt; 9 Department of Life Sciences, College of Science and Art Mahyel Aseer, King Khalid University, 62529 Abha, Saudi Arabia; 10 Unit of Food Bacteriology, Central Laboratory of Food Hygiene, Ministry of Health, Branch in Zagazig, Zagazig, 44511 Egypt; 11 Department of Pharmaceutical and Medicinal Chemistry, An der Immenburg 4, 53121 Bonn, Germany; 12 Department of Biological Sciences and Chemistry, University of Nizwa, Nizwa-616, Nizwa, Oman

**Keywords:** Medicinal chemistry, Organic chemistry

## Abstract

Type II diabetes mellitus (T2DM) is a global health issue with high rate of prevalence. The inhibition of *α*-glucosidase enzyme has prime importance in the management of T2DM. This study was established to synthesize Schiff bases of 1,3-dipheny urea (**3a–y**) and to investigate their in vitro anti-diabetic capability via inhibiting *α*-glucosidase, a key player in the catabolism of carbohydrates. The structures of all compounds were confirmed through various techniques including, Fourier-transform infrared spectroscopy (FTIR) and nuclear magnetic resonance (NMR) and mass-spectrometry (MS) methods. Interestingly all these compounds displayed potent inhibition IC_50_ values in range of 2.14–115 µM as compared to acarbose used as control. Additionally, all the compounds were docked at the active site of *α*-glucosidase to predict their mode of binding. The docking results indicates that Glu277 and Asn350 play important role in the stabilization of these compounds in the active site of enzyme. These molecules showed excellent predicted pharmacokinetics, physicochemical and drug-likeness profile. The anti-diabetic potential of these molecules signifies their medical importance and provide insights into prospective therapeutic options for the treatment of T2DM.

## Introduction

Diabetes mellitus type II is a globally health problem which has been considered a metabolic syndrome. Two possible reasons lack of enough insulin production or their proper action which leads to high blood glucose level. The primary causes of diabetes mellitus are excessive hepatic glucose production or glucose intolerance. The uncontrolled blood glucose concentration further resulting into severe consequences, like retinopathy, neuropathy, and nephropathy and as well other cardiovascular complications^1–4^.

*α*-Glucosidase (EC 3.2.1.20) is among a hydrolase group and thus inhibition of it suppress the glucose absorption resulting into a favourable effect over high blood glucose level. A crucial strategy for avoiding type II diabetes mellitus' deadly effects is to control blood glucose levels. Hence, there is an immense need to synthesize new small molecules and to evaluate their anti-diabetic potential against *α*-glucosidase, might be used as drug candidates for the treatment of type II diabetes mellitus^5–9^. Several therapeutic approaches of diabetes are available, but α-glucosidase inhibitors (AGIs), has been considered a precise and specific strategy for the management of type II diabetes mellitus. AGIs, have been considered a valuable approach because these AGIs slow down the catalytic activity of carbohydrates digestive enzyme *α*-glucosidase^10,11^.

Urea represents privileged structures that constitutes a crucial framework of a variety of drugs and bioactive compounds displaying broad range of diverse therapeutic and pharmacological properties. Various compounds with urea motifs are approved marketing drugs by various agencies like FDA. Indeed, they are promising drug candidates and represent a noteworthy place in position in academic research as well as synthetic and medicinal chemistry^12^.

Urea substituted with two aromatic moieties are thought to be Diarylureas, or bis-aryl ureas. Diarylurea is a significant scaffold embedded in different heterocyclic compounds with numerous pharmacological properties like antimalarial, antithrombotic, antitumor, anti-inflammatory, and antibacterial properties as a result they are widely used in drug discovery and drug design. Diaryl ureas have an excellent ability to bind with a variety of receptors^13^ and enzymes due to the presence of near perfect binding sites(NH) with acceptors (urea O) and as a result of this ability diarylureas display remarkable antitumor activities.

**Figure 1:**
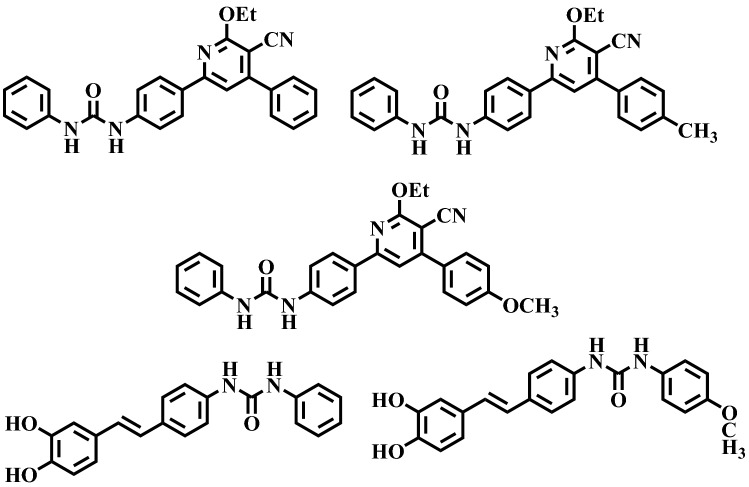
The available reported α-glucosidase inhibitors^18,19^.

Urea derivatives are well known anti-diabetic agents via targeting *α*-glucosidase enzyme. Several inhibitors have previously been discovered for this class demonstrating their potential for use in drug discovery^14–17^. Apart this phenyl urea comprising compounds and urea derivatives have been already reported for their anti-α-glucosidase inhibitory capability^18,19^ (Fig. 1).

Therefore, taking all these into consideration, we aimed to synthesize Schiff bases of 1,3-diphenyl urea derivatives **(3a–y)** by reacting urea derivatives with vanillin and substituted salicylic aldehyde, and their anti-α-glucosidase properties were investigated to explore their therapeutic role in the management of diabetes mellitus. In silico techniques have enormous applications in design and discovery of new and structurally diverse ligands which have high possibility to become drugs^20–26^. Based on excellent outcomes of docking method, we used docking to predict the binding modes of synthesized compounds in α-glucosidase.

## Results and discussion

### Chemistry

**Figure 2:**
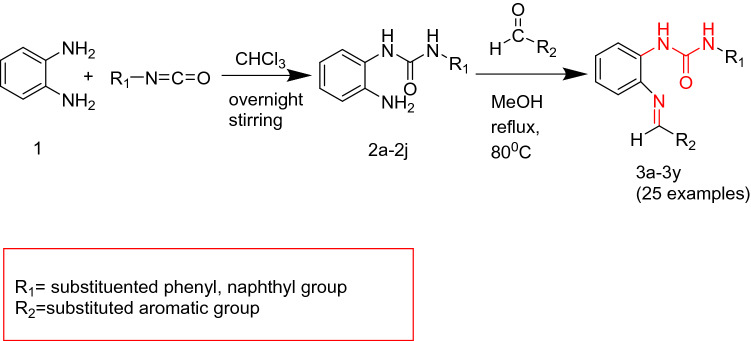
Synthesis of Schiff base 1, 3-dipheny urea derivatives.

Ortho phenylenediamine **(1)** was reacted with equimolar amount of different substituted isocyanates by constant stirring at room temperature overnight and resulting mono substituted 1,3-diphenyl ureas **(2a–j)** were refluxed for 3–4 h with substituted aldehydes via simple condensation by refluxing in methanol to obtain the final products **(3a–y)*.***The scope of reaction was broadened by using a variety of aldehydes including o-vanilline ,p-vanilline and 3-ethoxy salicylic aldehyde with different mono substituted 1,3-diphenyl ureas.The targeted compounds (**3a–y**) were obtained in good yields (**50–77%**) (Fig. 2).

The structures of the of schifff base 1,3-dipheny urea derivatives were established using microanalysis (CHN) and spectral data i.e., IR, 1H NMR, 13C NMR. The C=N band in FTIR appeared in the range of 1567–1614 cm^−1^. The ^1^H NMR peak that appeared in the range from *δ* 10–12 Ppm confirmed the presence of phenolic OH. HC=N and other peaks observed were also in accordance with the predicted structure. The spectral data of other aromatic and aliphatic protons were in accordance with these structures of anticipated compounds. In ESI spectra, the molecular ion peaks appeared as [M + H]+, which were in total agreement with the molecular weight of the synthesized compounds.

### Biology: in vitro* α*-glucosidase inhibitory activity

Total 25 synthetic derivatives of urea were evaluated against the key carbohydrates hydrolysing enzyme, *α*-glucosidase. All the compounds are active anti-*α*-glucosidase agents with varied potential due to the variation in their R substitution. These agents are categorized into group A, B, and C, according to variation in R_2_. In compounds **3a**–**3k**, R_1_ is diverse while R_2_ group is same (C_9_H_12_O_2_) which displayed potent *α*-glucosidase inhibitory capability (ranging from 3.96 to 45.55 µM, Table 1) as compared to marketed *α*-glucosidase inhibitor (AGI) (acarbose, IC_50_ = 875.41 ± 1.16 µM). Such as compound **3a**, *meta*-chloro group exhibited several folds more potent inhibition (IC_50_ = 5.84 ± 0.13 µM). In compound **3b**, *para*-flouro group substitution decreased its anti-diabetic activity (IC_50_ = 17.29 ± 0.18 µM), as compared to **3a**. In contrast similar flouro group substitution at *meta* position in **3c** enhanced its *α*-glucosidase inhibition (IC_50_ = 6.10 ± 0.12 µM), as compared to **3b**. In compound **3d**, naphthyl group substitution caused almost similar inhibitory potential like **3c**, with IC_50_ of 7.19 ± 0.15 µM. Compound **3e**, with *para*-methoxy substituent showed good anti-*α*-glucosidase potential (IC_50_ = 21.60 ± 0.30 µM). Unlike *meta*-chloro substitution in **3a**, the *para*-chloro substitution in **3f** decreased its *α*-glucosidase inhibitory capability (IC_50_ = 24.43 ± 0.31 µM).

**Table 1:** In vitro* α*-glucosidase inhibition of different substituents of Schiff bases of 1,3-diphenyl urea derivatives.

Comp	R_1_	R_2_	IC_50_ = ± SEM (µM)
Group A			
**3a**			5.84 ± 0.13
**3b**			17.29 ± 0.18
**3c**			6.10 ± 0.12
**3d**			7.19 ± 0.15
**3e**			21.60 ± 0.30
**3f**			24.43 ± 0.31
**3g**			18.43 ± 0.25
**3h**			3.96 ± 0.10
**3i**			16.37 ± 0.11
**3j**			45.55 ± 0.39
**3k**			23.11 ± 0.16
Group B			
**3l**			70.17 ± 1.34
**3m**			16.12 ± 0.20
**3n**			18.10 ± 0.35
**3o**			89.13 ± 0.52
**3p**			104.49 ± 0.60
**3q**			35.10 ± 0.27
**3r**			4.87 ± 0.13
**3s**			76.20 ± 0.51
**3t**			69.83 ± 0.74
**3u**			2.14 ± 0.11
**3v**			6.69 ± 0.20
Group C			
**3w**			85.37 ± 0.62
**3x**			115.94 ± 1.16
**3y**			88.56 ± 0.47
Standard: Acarbose (IC_50_ = 875.41 ± 1.16 µM)			

The effect of methyl substitution at *ortho* and *meta* positions on *α*-glucosidase inhibition was evaluated in compounds **3g** and **3h**, *meta*-methyl substituted **3h** (IC_50_ = 3.96 ± 0.10 µM) presented five times more potent inhibitory potential than **3g** (IC_50_ = 18.43 ± 0.25 µM). While *para*-methyl substituted compound **3i**, presented almost similar anti-diabetic potency (IC_50_ = 16.37 ± 0.11 µM), like ortho-methyl substituted compound (**3g**). On the other hand, addition of C_6_H_5_ in compound **3j**, declined its *α*-glucosidase inhibition (IC_50_ = 45.55 ± 0.39 µM). The addition of COCH_3_ in compound **3k** interestingly showed favourable anti-diabetic effect and enhanced the potency of **3k** (IC_50_ = 23.11 ± 0.16 µM).

In group B, compounds **3l**–**3v**, R_2_ group is same (C_8_H_10_O_2_), while R_1_ is varied. For instance, compound **3l** with *meta*-chloro group exhibited decreased inhibitory potential than **3a** (IC_50_ = 70.17 ± 1.34 µM) of group A. However, compound **3m** with *para*-flouro group exhibited almost same anti*-α*-glucosidase capability (IC_50_ = 16.12 ± 0.20 µM) like **3b** (group A). While in compound **3n**, the effect of *meta*-flouro was inverse (IC_50_ = 18.10 ± 0.35 µM) than group A compound **3c**. This inverse effect of variation in R_1_-group with C_8_H_10_O_2_ as R_2_ was also observed in **3o**–**3q**, which exhibited IC_50_ of 89.13 ± 0.52, 104.49 ± 0.60 and 35.10 ± 0.27 µM, respectively. Interestingly the anti-α-glucosidase activity of **3r** (IC_50_ = 4.87 ± 0.13 µM) is improved upon the addition of *ortho*-methyl-phenyl group at R_1_. However, *meta* and *para* substituted methyl phenyl ring substitution at R_1_ decreased the biological activity of **3s** (IC_50_ = 76.20 ± 0.51 µM) and **3t** (IC_50_ = 69.83 ± 0.74 µM), respectively. Surprisingly, the addition of phenyl ring in compound **3u** displayed extraordinary *α*-glucosidase inhibitory activity (IC_50_ = 2.14 ± 0.11 µM) and made it most potent agent of this series. Similarly, the substitution of *para*-COCH_3_-substituted phenyl in **3v**, also produced excellent effect on its *α*-glucosidase inhibitory action (IC_50_ = 6.69 ± 0.20 µM) than similar moity substituted compound (**3k**) in group A.

We have place three compounds (**3w**–**3y**) in group C, in which benzyl *ortho*-OH is replaced to *para* position at R_2_ and R_1_ is diverse. This positional change suppressed the *α*-glucosidase inhibitory potency of **3w**–**3y**, their biological activity was compared with the compounds with similar R_1_ moieties in group A and B. Compound **3w** showed lesser activity (IC_50_ = 85.37 ± 0.62 µM) than **3f** (group A) and** 3q** (group B). Similarly, **3x** (IC_50_ = 88.56 ± 0.47 µM) has lower potency than **3g** and **3r**, and the inhibitory activity of **3y** (IC_50_ = 115.94 ± 1.16 µM) is decreased than **3i** and **3t**. The structure–activity relationship (SAR) revealed that variation in R_1_ along with R_2_ displayed a key role in the inhibitory capability of *α*-glucosidase.

### In silico: analysis of binding mode by molecular docking

The active site of α-glucosidase comprises of three catalytic residues, Asp215, Glu277, and Asp352. Whereas several polar and hydrophobic residues including Asp69, Tyr72, Val109, His112, Phe159, Phe178, Gln182, Arg213, Asp215, Val216, His351, Arg442, and Arg446 surround those catalytic residues and contributes to the active site. The core of the active site is lined by a patch of hydrophobic residues that make grooves around the catalytic residues. We called these grooves as hydrophobic pocket 1 and 2.

Initially, acarbose was docked into the active site, which showed excellent interactions with the catalytic residues (Asp352, Asp215, and Glu277) and formed several hydrogen bonds with those residues. In addition, the polar moieties of acarbose also formed H-bonds with Asp69, Ser240 and several water molecules.

All the synthesized compounds showed inhibitory activity against α-glucosidase. Therefore, molecular docking was employed to determine the binding behaviour of these compounds within the active site of α-glucosidase. Seven compounds **3u**, **3h**, **3r, 3a**, **3c**, **3v** and **3d** exhibited highest inhibitory activities with IC_50_ values of 2.14 µM to 7.19 µM.

The docked conformation of most active compound, **3u** revealed that its urea moiety efficiently interacted with one of the catalytic residues of the active site, Glu277. Moreover, the side chain of Asn350 also provided hydrogen bond (H-bond) to the urea moiety. We observed that R_1_ of **3u** was inserted in the hydrophobic pocket 1 which is constituted by Trp58, Phe301, Tyr347, and Tyr387. These residues stabilize the cyclohexane ring through hydrophobic interaction. Additionally, the Glu277 provide hydrophobic (π-H) interaction to this phenyl ring of **3u**. Whereas the hydroxy-methoxy-phenyl ring (R_2_) was fitted at another hydrophobic pocket 2 which is composed of Tyr158, Phe159, Phe178, Val216, and Leu219. While the amino and urea linker phenyl ring resides at the entrance loop of the active site (Asp242, His280, Phe303, Asp307, Pro312, Phe314, Arg315, Tyr316, Glu411, and Asn415) and interact with these residues of entrance loop, therefore, block the access of the substrate in the active site. In addition, the -OH group of **3u** donated a H-bond to the side chain of Glu277. Due to these excellent binding interactions, **3u** produced highly negative docking score (DS = − 6.67 kcal/mol) in the binding region during docking.

The docked orientations of other most active hits, **3h**, **3r, 3a**, **3c**, **3v** and **3d** were similar to the docked conformation of **3u**, however, their amino-urea-linker phenyl ring twists more towards the surface of the active site, whereas their R_1_ and R_2_ substituted groups were placed in the hydrophobic pocket 1 and hydrophobic pocket 2, respectively. The urea moiety of **3h** accepted H-bond with the side chain of Asn350, while the urea of **3r** mediated multiple H-bonds with the side chains of Glu277, Asn350, and Asp352. Similarly, the urea of **3a** formed H-bonds with Asn350 and Asp352, moreover, the side chains of Tyr72, and Phe178 provide π-H interactions to the ethoxy group (R_2_) of **3a**. The binding modes of **3c** and **3v** depict that their urea forms H-bond with the side chain of Gln353, while –OH of **3c** (R_2_) interact with Glu277 through H-bond. Whereas the urea of **3d** interacts with the side chain of Glu411. It can be seen that the slight conformational difference can affect the binding modes of these compounds, thus alter their inhibitory activities.

Several compounds including **3m, 3i, 3b, 3n,** and **3g** exhibited IC_50_ in range of 16.12 to 18.43 µM. The binding modes of these compounds reflect that the urea moiety of **3m, 3i** and **3b** mediates only one H-bond with the side chain of Asp352. The R_2_ of **3m** and **3i** formed hydrophobic interaction with Glu277, while R_2_ of **3b** produced hydrophobic interaction with Tyr72 and Phe303. The amino-urea-phenyl linker of **3n** and **3g** was slipped more towards the entrance of the active site, due to this conformational change, their urea group interacted with the side chain of Asp307 at the entrance loop of the active site. Additionally, the -OH (at R_2_) of **3n** formed a H-bond with the side chain of Glu411 at the entrance loop of the active site. However, the R_2_ of **3g** did not interact with the surrounding residues.

The addition of bulky groups at R_1_ position is responsible to decrease the inhibitory potential of the compounds. It may be due to the steric hinderance caused by bulky moieties at R_1_ position in the hydrophobic pocket 1. Compounds **3e** (21.60 µM), **3k** (23.11 µM) **3f** (24.43 µM) also showed good inhibitory activities. The docked view of **3e** showed that its R_1_ and R_2_ groups did not interact with the surrounding residues in both hydrophobic pockets, while its urea formed a H-bond with the side chain of Asn350 near pocket 1. Interestingly, the docked orientation of **3k** was different from docked conformations of other compounds. The COCH_3_-phenyl ring (R_1_) of **3k** was oriented toward the entrance loop of the active site instead of hydrophobic pocket 1, due to this orientation, its urea group bent towards Asp352 and formed a H-bond with the side chain of Asp352 of catalytic triad. The compounds **3q** and **3j** exhibited moderate inhibitory activities in range of 35.10 to 45.55 µM. Compounds **3f** and **3q** adopted similar mode of binding like **3g**, and their urea also formed a H-bond with Asp307, additionally, those compounds mediated π–π interaction with Phe303 at the entrance loop of active site, and** 3q** further formed a methyl-π (hydrophobic) interaction with Arg315 of the loop. The docked view of **3j** revealed that its urea moiety did not form any interaction and adjusted towards the entrance of active site, while only its -OH group (R_2_) formed a H-bond with the side chain of Glu277.

**Figure 3:**
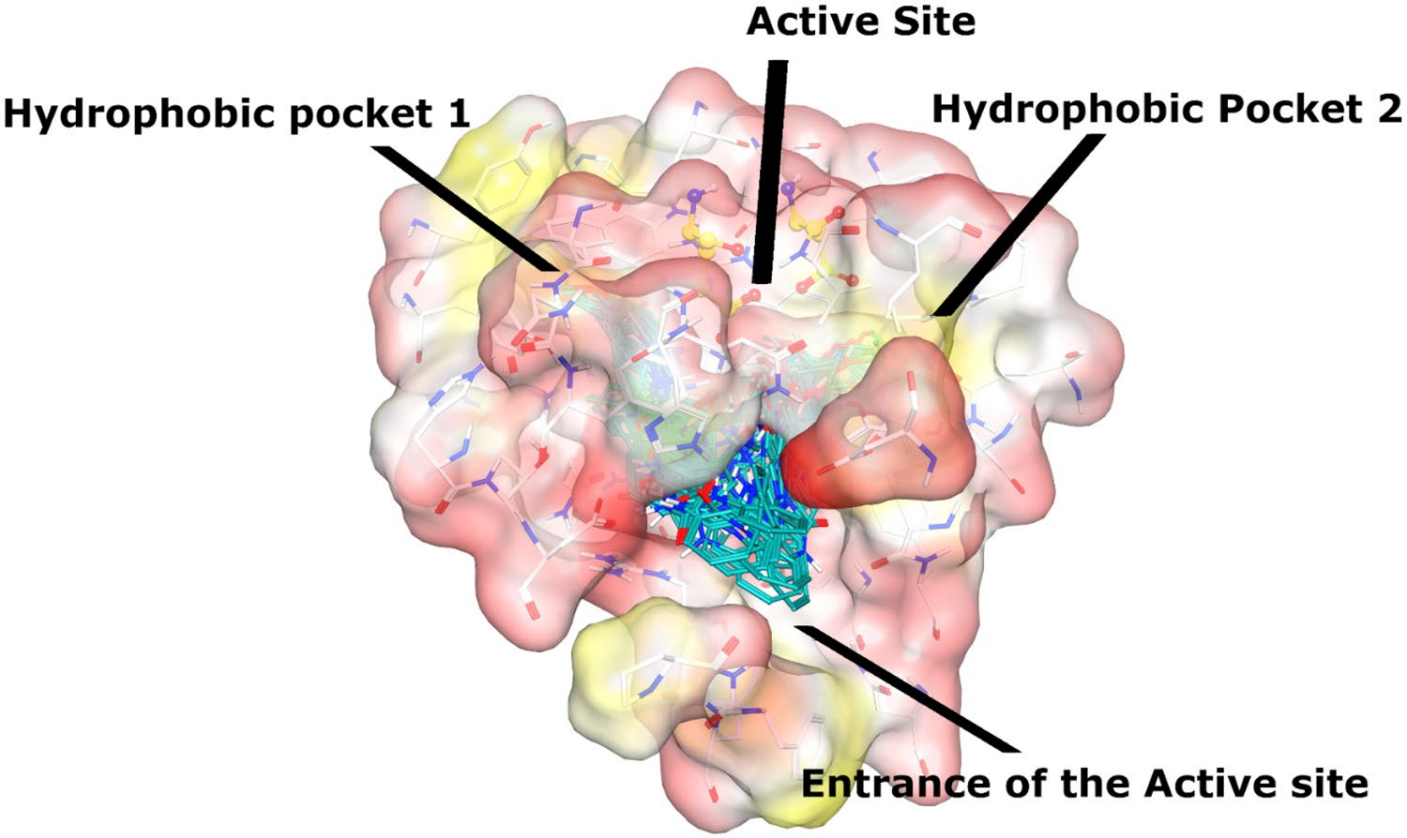
The docked view of all the compounds (shown in cyan stick model) is shown in the active site of α-glucosidase enzyme. The enzyme is presented in surface model where yellow and red colours shows hydrophobicity and hydrophilicity, respectively. The hydrophobic pocket 1 and 2 and the entrance loop of the active site shows hydrophobic behaviour. The catalytic residues are shown in yellow ball and stick model, while active site residues are depicted in white stick model.

**Figure 4:**
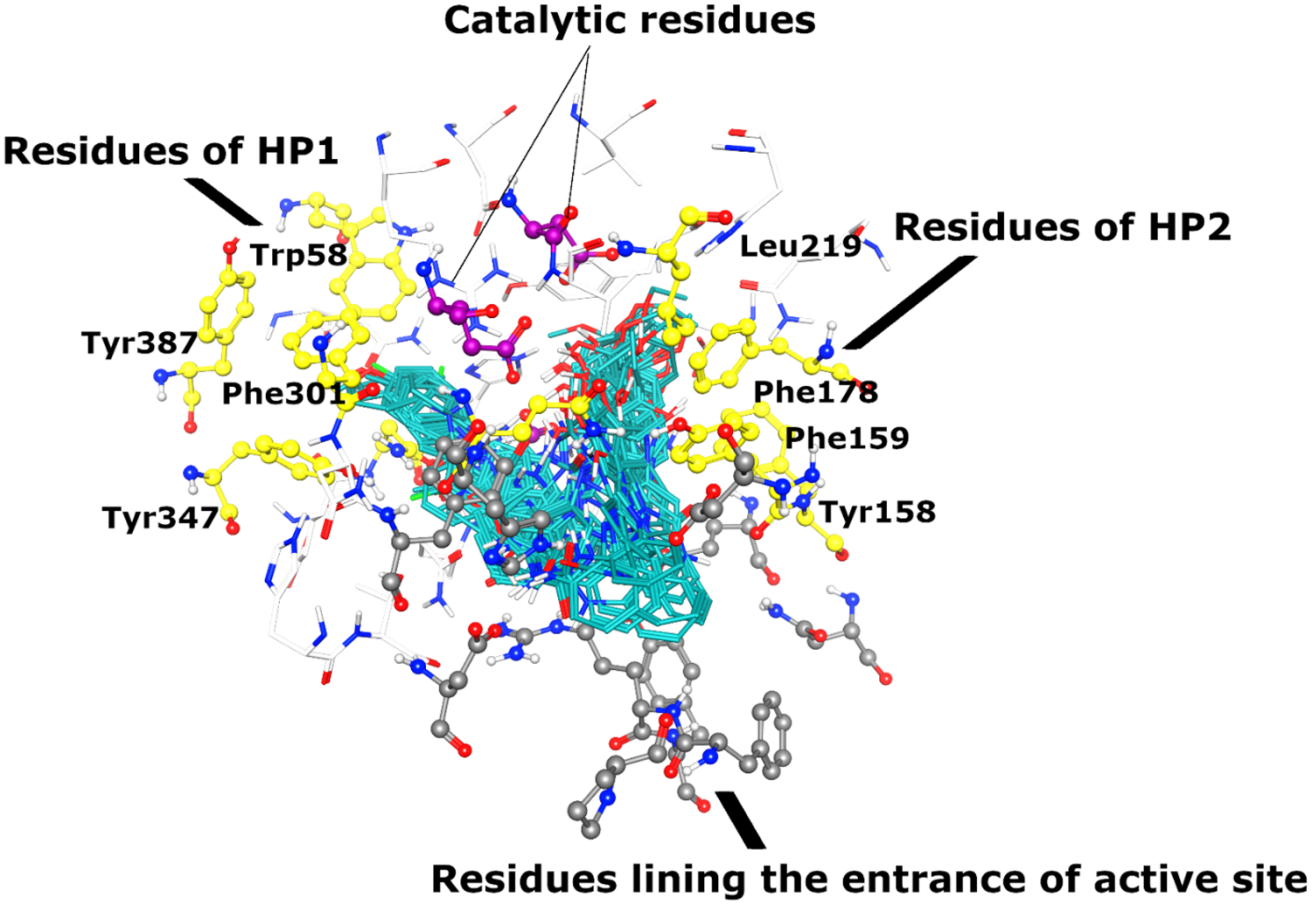
The docked orientation of all the compounds (cyan stick model) is shown with interacting residues. The residues of hydrophobic pocket 1 (HP1) and 2 (HP2) are shown in yellow ball and stick model. The residues of the active site entrance loop are shown in grey ball and stick model. The extended residues of active site are presented in white stick model.

**Figure 5:**
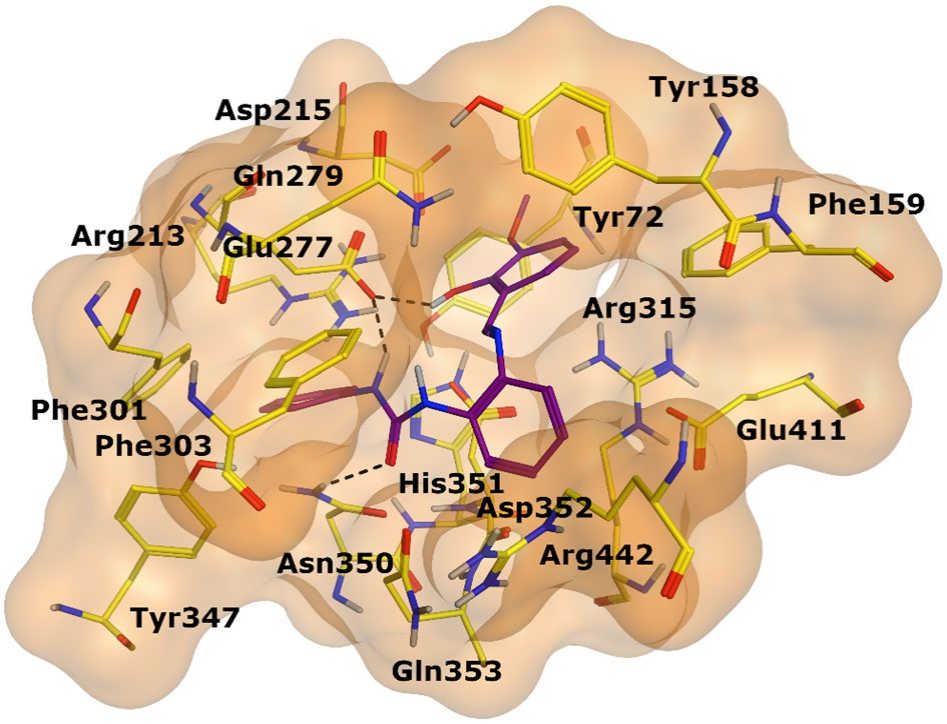
The binding mode of most active compound (**3u**) is shown in the active site of enzyme. **3u** is displayed in purple stick model, interacting residues are depicted in yellow stick model and Hydrogen bonds are shown in black dotted lines.

Several compounds including **3t**, **3l**, **3s**, **3w**, **3y**, **3o**, **3p**, and **3× **demonstrated moderate-to-least inhibitory potential (IC_50_ = 69.83 to 115.94 µM). The conformational analyses reflect that the bulky or steric groups at R_1_ position drags the compounds towards the entrance of active side instead of their interaction at the core of active site or in hydrophobic pockets. Due to the conformational changes, the R_1_/R_2_ moieties of these molecules do not fit properly in the hydrophobic pockets instead fits near the entrance loop. The methyl-benzene in **3t** adopted binding pattern like **3k**, the urea group of **3t** mediated H-bonding with Glu277, whereas it’s R_1_ moiety was tilted towards the entrance loop where formed π–π interaction with Phe303. Similarly, R_2_ of **3l** was twisted near the entrance loop where its urea donated a H-bond to Glu411, and its R_1_ formed π–π interaction with Phe303. The linker-phenyl ring of **3s** was surface exposed while its R_1_ group was bent towards loop, due to this bending its urea was oriented towards Asp307 at the entrance of the active site and formed a H-bond with Asp307. Similar binding mode was acquired by **3w** and **3y**, the urea moiety of **3w** and **3y** binds with His280 and Asp307, respectively which lines the entrance of the active site, and their R_2_ moiety forms H-bond with Glu277. The compound **3o** adopted such a conformation where its urea group face the catalytic triad and formed a H-bond with Asp352, while its linker ring and R_1_ moiety (naphthalene) face the entrance of the active site which makes π-π interaction with Phe303. Likewise, **3p** also mediated a H-bond with Asp352 through its urea group. The binding mode of **3× **revealed the reason of its least inhibitory potential. The amino and urea linker phenyl of **3× **was surface exposed and face the entrance of the active site, while its R_1_ was oriented towards the loop and mediates π-π interaction with Phe303, interestingly its R_2_ moiety slipped in the core of active site instead of fitting in hydrophobic pocket where it’s -OH formed a H-bond with Asp215. The docking results indicates that the addition of steric groups at R_1_ position produces conformational changes in compounds which are responsible for diverse biological activities of these compounds. The hydrophobic pockets and the binding mode of all the compounds are shown in Figs. 3 and 4. The docked view of most active compound (**3u**) is shown in the active site of α-glucosidase in Fig. 5. The enzyme-inhibitors binding interactions and the docking scores of each compound are tabulated in Table 2. The docking scores of compounds are in range of > − 6 to > − 2 kcal/mol, which indicates a good correlation with the in vitro results.

**Table 2:** The docking results of compounds** 3a–3y**.

Compounds	Score (kcal/mol)	Protein–ligand Interactions			
		Ligand atom	Enzyme atom	Interaction	Distance (Å)
**3a**	− 5.34	N5	OD2-ASP352	HBD	2.74
		O2	ND2-ASN350	HBA	2.03
		C43	6-ring-PHE178	Π-H	3.03
**3b**	− 4.75	N5	OD2-ASP352	HBD	2.04
		C35	6-ring TYR72	Π-H	3.32
		6-ring	6-ring PHE303	Π-Π	3.27
**3c**	− 5.22	N5	OE1-GLN353	HBD	2.58
		O39	OE2-GLU277	HBD	2.42
		6-ring	6-ring-PHE178	Π-Π	3.90
**3d**	− 5.19	N3	OE2-GLU411	HBD	2.23
		6-ring	6-ring-PHE303	Π-Π	3.98
		6-ring	6-ring-PHE303	Π-Π	3.06
**3e**	− 4.37	O2	ND2-ASN350	HBA	2.51
**3f**	− 4.14	N3	OD1-ASP307	HBD	2.52
		6-ring	6-ring-PHE303	Π-Π	3.47
**3g**	− 4.60	N3	OD1-ASP307	HBD	2.17
		6-ring	6-ringPHE303	Π-Π	3.53
**3h**	− 6.53	O2	ND2-ASN350	HBA	2.12
**3i**	− 4.84	N5	OD2-ASP352	HBD	2.03
		6-ring	CG-GLU277	Π-H	2.99
**3j**	− 3.87	O40	OE2-GLU277	HBD	1.92
**3k**	− 4.14	N5	OD2-ASP352	HBD	2.17
**3l**	− 3.58	N3	OE2-GLU411	HBD	2.05
		6-ring	6-ring-PHE303	Π-Π	3.91
**3m**	− 4.87	N5	OD2-ASP352	HBD	2.19
		6-ring	CG-GLU277	Π-H	2.96
**3n**	− 4.54	N5	OD1-ASP307	HBD	2.41
		O39	OE2-GLU411	HBD	2.35
**3o**	− 3.64	N5	OD2-ASP352	HBD	2.71
		6-ring	6-ring-PHE303	Π-Π	3.96
		6-ring	6-ring-PHE303	Π-Π	3.96
**3p**	− 2.96	N5	OD2-ASP352	HBD	2.97
**3q**	− 3.95	N3	OD1-ASP307	HBD	2.25
		6-ring	CB-ARG315	Π-H	3.99
		6-ring	6-ring-PHE303	Π-Π	3.45
**3r**	− 6.50	N3	OD2-ASP352	HBD	2.41
		N5	OE2-GLU277	HBD	2.19
		O39	OE2-GLU277	HBD	2.12
		O2	ND2-ASN350	HBA	1.90
		6-ring	6-ring PHE303	Π-Π	3.93
		6-ring	6-ring PHE301	Π-Π	3.49
**3s**	− 3.33	N5	OD1-ASP307	HBD	2.89
**3t**	− 3.67	N5	OE2-GLU277	HBD	2.90
		6-ring	6-ring PHE303	Π-Π	3.25
**3u**	− 6.67	N5	OE2-GLU277	HBD	1.98
		O40	OE2-GLU277	HBD	1.99
		O2	ND2-ASN350	HBA	2.39
		6-ring	CG-GLU277	Π-H	3.96
**3v**	− 5.08	N3	NH1-ARG442	HBA	3.06
		6-ring	CG-GLU277	Π-H	3.90
		6-ring	CB-ASP352	Π-H	3.33
**3w**	− 3.37	O40	OE2-GLU277	HBD	2.56
		O2	NE2-HIS280	HBA	1.91
**3x**	− 2.81	O43	OD1-ASP215	HBD	2.09
		6-ring	6-ring-PHE303	Π-Π	3.20
**3y**	− 3.26	N5	OD1-ASP307	HBD	2.91
		O43	OE1-GLU277	HBD	3.0
		O43	OE2-GLU277	HBD	2.99
		6-ring	6-ring PHE303	Π-Π	3.67
**Acarbose**	− 4.59	O5	OE2-GLU277	HBD	2.88
		O14	OD1-ASP352	HBD	2.69
		O16	OD2-ASP69	HBD	2.64
		O20	OD1-ASP215	HBD	2.93
		O20	OD2-ASP215	HBD	2.81
		O24	OG-SER240	HBD	2.71
		O28	OD2-ASP242	HBD	2.93

### In silico ADMET Calculation

The ADMET (Absorption, Distribution, Metabolism, Excretion and Toxicity) was predicted through computational tool, SwissADME which shows good physicochemical, pharmacokinetic profile and drug like and medicinal chemistry properties of these compounds. The molecular weight of all the compounds is < 500 with number of rotatable bonds in range of 7–9, number of hydrogen bond donor and acceptors in range of 3, and 4–5, respectively. The synthesized hits have topological polar surface area values of 82.95–100.02Å^2^ with partition coefficient (logP_o/w_) of 2.82–4.09, which suggest that these compounds have low to good solubility in lipid bilayer. While these compounds showed moderated water solubility (Table S1).

The predicted pharmacokinetic (Table S2) profile of 3a–3y reflect these compounds have high gastrointestinal absorption, and no blood brain barrier permeability and substrate likeness for P-glycoprotein, therefore, these molecules are safe. Similarly, their ability to permeate skin is also low. Similarly, these molecules follow all the drug-likeness rules of Lipinski rule of five, and all the compounds (except **3d**) did not show any violation of Ghose, Veber, Egan and Muegge’s rules of drug-likeness. Their bioavailability and synthetic accessibility scores indicate that these compounds are moderately bio-available, and synthetic feasible. The predicted ADMET profile reflect that these molecules can serve as good drug candidates upon further optimization.

### Experimental work

#### Materials and method

All the starting materials employed in the synthesis were purchased from Sigma-Aldrich Co. (Germany) and used without purification. Methanol, absolute ethanol, and other solvents were also purchased from different commercial sources in adequate purity and used without purification in the reaction media. To monitor the reaction, thin layer chromatography (TLC) was performed with silica gel 60 aluminum-backed plates with suitable solvent system. Spotson TLC plated were visualized by using the UV light with 254 nm. The infrared (IR) spectra were recorded in the range of 400–4000 cm-1 on IR Affinity-I (Shimadzu) spectrophotometer. The ^1^H and ^13^C nuclear magnetic resonance (NMR) spectra were recorded using DMSO-d6 andCDCl3 as solvents via Bruker spectrophotometer 300, 400 and 600 MHz as dilute solution at 25 °C. Chemical shifts were reported in parts per million (δ = ppm) and coupling constants (J) were expressed in Hertz (Hz). The signals were described as singlet (s), doublet (d), triplet (t) multiplet (m). Mass spectra (ESI–MS), in turn, were recorded by means of Bruker Daltonics mass spectrometer. Melting points were determined on cover slips using Stuart melting point apparatus and are uncorrected.

#### Chemistry: general procedure for the synthesis of Schiff base 1,3-dipheny urea derivatives

Ortho phenylenediamine **(1)** (5 mmol) was dissolved in 15–20 ml of chloroform by constant stirring at room temperature. Then equimolar amount of different substituted isocyanates added carefully dropwise with the help of dropping funnel into this diamine solution. Immediately, solid product precipitated out at stirring that was filtered followed by washing with n-hexane and dried under vacuum. The resulting mono substituted 1,3-diphenyl urea **(2a–j)** (1 mmol) were refluxed for 3–4 h with substituted aldehydes (1 mmol) in 8–10 mL of methanol to obtain the final products **(3a–y)** that were filtered, washed with cold ethanol, and dried under vacuum.

##### (E)-1-(3-chlorophenyl)-3-(2-((3-ethoxy-2-hydroxybenzylidene) amino) phenyl) urea (3a)

Yellow solid; Yield: 55%, m.p: 218–220 °C; IR ʋ max (cm^−1^): 3300 (NH), 1613 (C=N), 1649(C=O), ^1^H-NMR (DMSO-*d*^6^) δ ppm;1.34 (t, 3H, CH_3,*,*_*J* = 6.6 Hz), 4.07(q,2H, CH_2_ *,J* = 6.6 Hz), 6.91(t,1H,* J* = 7.8 Hz), 7.01 (d,1H,* J* = 7.8 Hz), 7.09 (t,1H,* J* = 7.2 Hz), 7.14 (d,1H,* J* = 7.8 Hz), 7.22–7.30 (m,4H), 7.39 (d,1H,* J* = 7.2 Hz), 7.73 (s,1H), 8.04 (d,1H,* J* = 7.8 Hz),8.27 (s,1H),8.89 (s,1H),9.54 (s,1H), 11.91(s,1H); ^13^C-NMR ppm;14.7, 64.1, 116.6, 116.7, 117.6, 118.8, 118.9, 120.3, 120.5, 121.5, 123.2, 123.3, 127.0, 130.4, 132.8, 133.2, 139.2, 141.2, 147.9, 152.2, 163.1; C_22_H_20_ClN_3_O_3_ (409.12) m/z (%): 410.11[M + H] + (100).

##### (E)-1-(2-((3-ethoxy-2-hydroxybenzylidene) amino) phenyl)-3-(4-fluorophenyl) urea (3b)

Yellow solid; Yield: 54%, m.p: 217–219 °C; IR ʋ max (cm^−1^): 3299 (NH), 1614 (C=N), 1650(C=O), ^1^H-NMR (DMSO-*d*^6^) δ ppm;1.35 (t, 3H, CH_3_,* J* = 6.6 Hz), 4.07(q,2H, CH_2_,* J* = 6.6 Hz), 6.91(t,1H,* J* = 7.8 Hz), 7.06–7.14 (m,4H), 7.22–7.26 (m,2H), 7.38 (d,1H,* J* = 7.8 Hz), 7.46(s,1H), 8.07(d,1H,* J* = 7.8 Hz), 8.19(s,1H), 8.88(s,1H), 9.38(s,1H), 11.938(s,1H); ^13^C- NMR δ ppm;14.7, 64.1, 115.4, 116.7, 118.8, 120.0, 120.3, 122.9, 123.3, 127.0, 133.1, 136.0, 138.9, 147.1, 149.9, 152.4, 156.6, 158.1, 163.1; C_22_H_20_FN_3_O_3_ (393.42) m/z (%): 394.13 [M + H] + (100).

##### (E)-1-(2-((3-ethoxy-2-hydroxybenzylidene) amino) phenyl)-3-(3-fluorophenyl) urea (3c)

Yellow solid; Yield: 55%, m.p: 219–221 °C; IR ʋ max (cm^−1^): 2981 (NH), 1591 (C=N), 1650(C=O), ^1^H-NMR (DMSO-*d*^6^) δ ppm; 1.34(t, 3H, CH_3_,* J* = 6.6 Hz), 4.07(q,2H, CH_2_* J* = 6.6 Hz), 6.77(t,1H,* J* = 8.4 Hz), 6.91(t,1H,* J* = 7.2 Hz), 7.09t,1H,* J* = 6.6 Hz), 7.14(d,1H,* J* = 7.8 Hz), 7.23–7.31(m,3H), 7.38(d,1H,* J* = 7.8 Hz), 7.5(d,1H,* J* = 12 Hz), 8.04(d,1H,* J* = 8.4 Hz), 8.27(s,1H),8.89 (s,1H), 9.56(s,1H),11.93 (s,1H); ^13^C- NMR δ ppm;14.7, 64.1, 104.8, 105.0, 108.1, 108.3, 113.9, 116.7, 118.8, 120.3, 120.5, 123.3, 127.0, 130.4, 139.2, 141.6, 147.1, 149.9, 152.2, 161.6, 163.2; C_22_H_20_FN_3_O_3_ (393.42) m/z (%): 394.13 [M + H] + (100).

##### (E)-1-(2-((3-ethoxy-2-hydroxybenzylidene) amino) phenyl)-3-(naphthalen-2-yl) urea (3d)

Yellow solid; Yield: 60%, m.p: 228–230 °C; IR ʋ max (cm^−1^): 2973 (NH), 1554 (C=N), 1650 (C=O), ^1^H-NMR (DMSO-*d*^6^) δ ppm; 1.33 (t, 3H, CH_3_,* J* = 6.6 Hz), 4.08(q,2H, CH_2_* J* = 6.6 Hz), 6.92(t,1H,* J* = 7.2 Hz), 7.10(t,1H,* J* = 7.2 Hz), 7.14(d,1H,* J* = 7.8 Hz), 7.26(t,1H,* J* = 7.2 Hz), 7.31(d,1H,* J* = 7.8 Hz), 7.35(d,1H,* J* = 7.8 Hz), 7.47(t,1H,* J* = 7.8 Hz), 7.53(t,1H,* J* = 7.2 Hz), 7.57(t,1H,* J* = 6.0 Hz), 7.65(d,1H,* J* = 8.4 Hz), 7.89–7.93(m,2H), 8.04(d,1H,* J* = 8.4 Hz), 8.13(d,1H,* J* = 8.4 Hz), 8.61(s,1H), 8.93(s,1H), 9.32(s,1H), 12.22(s,1H); ^13^C- NMR δ ppm;14.7, 64.1, 116.8, 118.7, 118.8, 118.9, 120.2, 121.0, 121.8, 123.1, 123.4, 123.5, 125.6, 125.8, 125.9, 126.5, 127.0, 128.3, 133.2, 133.7, 134.2, 139.2, 147.1, 150.1, 153.0, 163.3; C_26_H_23_N_3_O_3_ (425.49) m/z (%): 426.17 [M + H] + (100).

##### (E)-1-(2-((3-ethoxy-2-hydroxybenzylidene) amino) phenyl)-3-(4-methoxyphenyl) urea (3e)

Orange Yellow solid; Yield: 59%, m.p: 204-206 °C; IR ʋ max (cm-1): 2973 (NH), 1612 (C=N), 1646(C=O), ^1^H-NMR (DMSO-d6) δ ppm; 1.35 (t, 3H, CH_3_,* J* = 7.2 Hz), 3.70 (s,3H), 4.08(q,2H, CH_2_,* J* = 6.6 Hz), 6.85 (d,2H,* J* = 7.8 Hz), 6.91(t,1H,* J* = 7.8 Hz), 7.050(t,1H,* J* = 7.2 Hz), 7.13 (d,1H,* J* = 7.8 Hz), 7.21–7.24 (m,2H), 7.34–7.37 (m,3H), 8.09(d,1H,* J* = 8.4 Hz), 8.12 (s,1H), 8.88(s,1H), 9.16 (s,1H), 11.92(s,1H); ^13^C- NMR δ ppm; 14.7, 55.1, 64.1, 114.0, 116.7, 118., 118.9, 120.1, 120.3, 122.6, 123.3, 127.0, 132.6, 133.4, 138.7, 147.1, 149.9, 152.5, 154.5, 163.0; C_23_H_23_N_3_O_4_ (405.45) m/z (%): 406.16 [M + H] + (100).

##### (E)-1-(4-chlorophenyl)-3-(2-((3-ethoxy-2-hydroxybenzylidene) amino) phenyl) urea (3f)

Yellow solid; Yield: 61%, m.p: 218–220 °C; IR ʋ max (cm^−1^): 2980 (NH), 1616 (C=N), 1649(C=O), ^1^H-NMR (DMSO-*d*^6^) δ ppm; 1.34 (t, 3H, CH_3_,* J* = 6.6 Hz), 4.07(q,2H, CH_2_,* J* = 6.6 Hz), 6.91(t,1H,* J* = 7.8 Hz), 7.08(t,1H,* J* = 7.2 Hz), 7.13 (d,1H,* J* = 7.8 Hz), 7.22–7.27 (m,2H), 7.31 (d,2H,* J* = 8.4 Hz), 7.38 (d,1H,* J* = 7.8 Hz), 7.48 (d,2H,* J* = 8.4 Hz), 8.06(d,1H,* J* = 7.8 Hz), 8.24(s,1H), 8.89 (s,1H), 9.48 (s,1H), 11.93(s,1H); ^13^C- NMR δ ppm;14.7,64.1, 116.7, 118.8, 118.9, 119.7, 120.3, 120.4, 123.0, 123.3, 125.4, 127.0, 128.7, 133.0, 138.7, 139.0, 147.1, 149.9, 152.2, 163.1; C_22_H_20_N_3_O_3_ (409.12) m/z (%): 410.11[M + H] + (100).

##### (E)-1-(2-((3-ethoxy-2-hydroxybenzylidene) amino) phenyl)-3-(o-tolyl) urea (3g)

Orange Yellow solid; Yield: 65%, m.p: 216-218 °C; IR ʋ max (cm^−1^): 3301 (NH), 1614 (C=N), 1649(C=O), ^1^H-NMR (DMSO-d6) δ ppm; 1.34 (t, 3H, CH_3_,* J* = 6.6 Hz), 2.23 (s,3H), 4.07(q,2H, CH_2_,* J* = 6.6 Hz), 6.90(t,1H,* J* = 7.8 Hz), 6.96(t,1H,* J* = 7.8 Hz), 7.08(t,1H,* J* = 7.8 Hz), 7.12(t,2H,* J* = 7.8 Hz), 7.16(d,1H,* J* = 7.8 Hz), 7.23(t,1H,* J* = 7.8 Hz), 7.28(d,1H,* J* = 7.8 Hz), 7.34(d,1H,* J* = 7.8 Hz), 7.65(d,1H,* J* = 7.8 Hz), 7.97(d,1H,*J* = 7.8 Hz), 8.50 (s,2H), 8.90(s,1H), 12.20(s,1H); ^13^C- NMR δ ppm;14.7,18.9,64.1, 116.8, 118.8, 120.2, 121.2, 122.5, 123.0, 123.2, 123.5, 126.1, 127.0, 128.7, 130.2, 133.3, 137.1, 139.2, 147.0, 150.1, 152.8, 163.0; C_23_H_23_N_3_O_3_ (389.46) m/z (%): 390.17 [M + H] + (100) 00%, m.p: 200–200 °C; IR ʋ max (cm-1): 2973 (NH), 1591 (C=N), 1647(C=O), 1H-NMR (DMSO-d6) δ ppm; (t, 3H, CH3), (q,2H, CH2), (d,1H), (s,1H), (s,1H), (s,1H), (s,1H); 13C NMR δ ppm;C_23_H_23_N_3_O_3_ (389.46) m/z (%): 390.17 [M + H] + (100).

##### (E)-1-(2-((3-ethoxy-2-hydroxybenzylidene) amino) phenyl)-3-(m-tolyl) urea (3h)

Cream Yellow solid; Yield: 51%, m.p: 203–205 °C; IR ʋ max (cm-1): 3301 (NH), 1614 (C=N), 1649(C=O), ^1^H-NMR (DMSO-d6) δ ppm; 1.35 (t, 3H, CH_3_,* J* = 6.6 Hz), 2.26 (s,3H), 4.08(q,2H, CH_2_,* J* = 6.6 Hz), 6.78 (d,1H,* J* = 7.2 Hz), 6.91(t,1H,* J* = 7.8 Hz), 7.06(t,1H,* J* = 7.8 Hz), 7.14(t,2H,* J* = 7.8 Hz), 7.22–7.26 (m,3H), 7.30 (s,1H), 7.37 (d,1H,* J* = 7.8 Hz), 8.08(d,1H, *J* = 7.8 Hz), 8.20(s,1H), 8.88 (s,1H), 9.27(s,1H), 11.94(s,1H); ^13^C- NMR δ ppm;14.7, 21.2, 64.1, 115.4, 116.7, 118.8, 118.9, 120.2, 120.3, 122.6, 122.8, 123.3, 127.0, 128.7, 133.2, 138.0, 138.9, 139.6, 147.1, 149.9, 152.3, 163.1; C_23_H_23_N_3_O_3_ (389.46) m/z (%): 390.17 [M + H] + (100).

##### (E)-1-(2-((3-ethoxy-2-hydroxybenzylidene)amino)phenyl)-3-(p-tolyl)urea (3i)

Cream Yellow solid; Yield: 52%, m.p:210–212 °C; IR ʋ max (cm-1): 3301 (NH), 1615 (C=N), 1649(C=O), ^1^H-NMR (DMSO-d6) δ ppm; 1.35 (t, 3H, CH_3_,* J* = 6.6 Hz), 2.23 (s,3H), 4.08(q,2H, CH_2_,* J* = 6.6 Hz), 6.91(t,1H,* J* = 7.8 Hz), 7.04–7.08 (m,3H), 7.13 (d,1H,* J* = 7.8 Hz), 7.21–7.25 (m,2H),7.33(d,1H,*J* = 7.8 Hz),7.38(d,1H,*J* = 7.8 Hz),8.09(d,1H,*J* = 7.8 Hz),8.17(s,1H),8.88(s,1H),9.25(s,1H),11.94(s,1H); ^13^C- NMR δppm;14.7, 20.3, 64.1, 116.7, 118.2, 118.3, 118.8, 120.1, 120.3, 122.7, 123.3, 127.0, 129.2, 130.7, 133.3, 137.1, 138.8, 147.1, 149.9, 152.3, 163.1; C_23_H_23_N_3_O_3_ (389.46) m/z (%): 390.17 [M + H] + (100).

##### (E)-1-(2-((3-ethoxy-2-hydroxybenzylidene) amino) phenyl)-3-phenylurea (3j)

Yellow solid; Yield: 55%, m.p: 200–202 °C; IR ʋ max (cm^−1^): 2980 (NH), 1616 (C=N), 1649(C=O), ^1^H-NMR (DMSO-*d*^6^) δ ppm; 1.35 (t, 3H, CH_3_,* J* = 6.6 Hz), 4.07(q,2H, CH_2_,* J* = 6.6 Hz), 6.91(t,1H,* J* = 7.8 Hz), 6.96(t,1H,* J* = 7.2 Hz), 7.05–7.08 (m,1H), 7.14 (dd,1H,* J* = 8.4,1.2 Hz), 7.22–7.28 (m,4H), 7.38 (dd,1H,* J* = 7.8,1.2 Hz), 7.46 (d,2H,* J* = 7.2 Hz), 8.09(dd,1H,* J* = 7.8,0.6 Hz), 8.22(s,1H),8.89 (s,1H),9.35 (s,1H), 11.96(s,1H); ^13^C- NMR δ ppm;14.8, 64.1, 116.7, 118.3, 118.9, 120.3, 121.9, 122.9, 123.4, 127.0, 128.9, 133.2, 138.9, 139.7, 147.1, 150.0, 152.4, 163.2; C_22_H_21_N_3_O_3_ (375.16) m/z (%): 376.11[M + H] + (100).

##### (E)-1-(4-acetylphenyl)-3-(2-((3-ethoxy-2-hydroxybenzylidene) amino) phenyl) urea (3k)

Yellow solid; Yield: 62%, m.p: 209–211 °C; IR ʋ max (cm-1): 3299 (NH), 1614 (C=N), 1650 (C=O), ^1^H-NMR (DMSO-d6) δ ppm; 1.34 (t, 3H, CH_3_,* J* = 7.2 Hz), 3.31 (s,3H), 4.07(q,2H, CH_2_,* J* = 7.2 Hz), 6.91(t,1H,* J* = 7.8 Hz), 7.10(t,1H,* J* = 7.8 Hz), 7.14 (d,1H,* J* = 7.8 Hz), 7.24–7.28 (m,2H), 7.39 (d,1H,* J* = 7.8 Hz), 7.59(d,2H,* J* = 7.8 Hz), 7.90 (d,2H,* J* = 7.8 Hz), 8.07(d,1H,* J* = 7.8 Hz), 8.36(s,1H), 8.90(s,1H),9.75 (s,1H),11.91 (s,1H); ^13^C- NMR δ ppm;14.7, 26.3, 64.1, 116.7, 117.2, 118.9, 118.9, 120.3, 120.5, 123.3, 127.0, 129.7, 130.5, 132.7, 139.2, 144.3, 147.1, 149.9, 152.0, 163.2, 196.3; C_24_H_23_N_3_O_4_ (417.47) m/z (%): 418.16) [M + H] + (100).

##### (E)-1-(3-chlorophenyl)-3-(2-((2-hydroxy-3-methoxybenzylidene) amino) phenyl) urea (3l)

Orange Yellow solid; Yield: 54%, m.p: 208–210 °C; IR ʋ max (cm^−1^): 3300 (NH), 1613 (C=N), 1649(C=O), ^1^H-NMR (DMSO-*d*^6^) δ ppm;3.82(s,3H, CH_3_) 6.93(t,1H,* J* = 7.8 Hz), 7.01 (d,1H,* J* = 7.8 Hz), 7.09 (t,1H,* J* = 7.2 Hz), 7.15 (d,1H,* J* = 7.8 Hz), 7.22–7.30 (m,4H), 7.42 (d,1H,* J* = 7.8 Hz), 7.72 (s,1H), 8.04 (d,1H,* J* = 8.4 Hz),8.29 (s,1H),8.89 (s,1H),9.54 (s,1H), 11.91(s,1H); ^13^C NMR ppm; 55.9, 115.5, 116.6, 117.6, 118.2, 118.9, 120.4, 121.5, 122.9, 123.2, 127.0, 130.4, 132.8, 133.2, 139.2, 141.2, 148.0, 149.6, 152.2, 162.5; C_21_H_18_ClN_3_O_3_ (395.84) m/z (%): 396.10[M + H] + (100).

##### (E)-1-(4-fluorophenyl)-3-(2-((2-hydroxy-3-methoxybenzylidene) amino) phenyl) urea (3m)

Yellow solid; Yield: 54%, m.p: 119–200 °C; IR ʋ max (cm^−1^): 3300 (NH), 1613 (C=N), 1649(C=O), ^1^H-NMR (DMSO-*d*^6^) δ ppm; 3.83(s,3H,CH_3_), 6.93(t,1H,* J* = 7.8 Hz), 7.05–7.12 (m,3H), 7.15(d,1H,* J* = 7.8 Hz), 7.21–7.25 (m,2H), 7.40–7.46 (m,3H), 8.06 (d,1H,* J* = 8.4 Hz), 8.21(s,1H), 8.89(s,1H), 9.36(s,1H), 11.83(s,1H); ^13^C- NMR ppm; 56.0, 115.3, 115.5, 118.9, 120.0, 120.4, 122.9, 123.0, 127.0, 133.2, 136.0, 139.0, 148.0, 149.7, 152.4, 156.6, 158.2, 162.5; C_21_H_18_FN_3_O_3_ (379.39) m/z (%): 380.13[M + H] + (100).

##### (E)-1-(3-fluorophenyl)-3-(2-((2-hydroxy-3-methoxybenzylidene) amino) phenyl) urea (3n)

Yellow solid; Yield: 50%, m.p: 200–201 °C; IR ʋ max (cm^−1^): 3300 (NH), 1613 (C=N), 1649(C=O), ^1^H-NMR (DMSO-*d*^6^) δ ppm; 3.83(s,3H,CH_3_), 6.77 (t,1H,* J* = 8.4 Hz), 6.93(t,1H,* J* = 7.8 Hz), 7.08(t,2H,* J* = 8.4 Hz), 7.15 (d,1H,* J* = 7.8 Hz), 7.23–7.30 (m,3H), 7.41(d,1H,* J* = 7.2 Hz), 7.50(d,1H,* J* = 12 Hz), 8.06 (d,1H,* J* = 8.4 Hz),8.30 (s,1H),8.90(s,1H),9.57(s,1H), 11.83(s,1H); ^13^C- NMR ppm; 56.0, 104.8, 105.0, 108.3, 113.9, 115.5, 118.9, 120.4, 123.0, 123.2, 127.0, 130.4, 132.9, 139.2, 141.6, 148.0, 148.7, 152.2, 161.7, 162.6, 163.3; C_21_H_18_FN_3_O_3_ (379.39) m/z (%): 380.13[M + H] + (100).

##### (E)-1-(2-((2-hydroxy-3-methoxybenzylidene) amino) phenyl)-3-(naphthalen-2-yl) urea (3o)

Orange Yellow solid; Yield: 77%, m.p: 214–216 °C; IR ʋ max (cm^−1^): 3300 (NH), 1613 (C=N), 1649(C=O), ^1^H-NMR (DMSO-*d*^6^) δ ppm; 3.83(s,3H,CH_3_) 6.94(t,1H,* J* = 7.8 Hz), 7.10(t,1H,* J* = 7.2 Hz), 7.15(d,1H,* J* = 7.8 Hz), 7.25(t,1H,* J* = 7.8 Hz), 7.28(d,1H,* J* = 7.8 Hz), 7.37(d,1H,* J* = 7.8 Hz), 7.47–7.58(m,3H), 7.65(d,1H,* J* = 8.4 Hz),7.92(t,2H,* J* = 6.6 Hz), 8.07(d,1H,* J* = 8.4 Hz), 8.13(d,1H,* J* = 8.4 Hz), 8.64(s,1H), 8.94(s,1H), 9.32(s,1H), 12.11(s,1H); ^13^C-NMR δ ppm;56.0,115.6,118.8,118.9,120.4,120.9,121.9, 123.1, 123, 2, 123.4, 125.7, 125, 9, 126.0, 126.5, 127.1, 128.4, 133.3, 133.8, 134.2, 139.2, 148.0, 149.8, 153.1, 162.8; C_25_H_21_N_3_O_3_ (411.46) m/z (%): 412.16[M + H] + (100).

##### (E)-1-(2-((2-hydroxy-3-methoxybenzylidene) amino) phenyl)-3-(4-methoxyphenyl) urea (3p)

Yellow solid; Yield: 52%, m.p: 204–206 °C; IR ʋ max (cm^−1^): 3300 (NH), 1613 (C=N), 1649(C=O), ^1^H-NMR (DMSO-*d*^6^) δ ppm; 3.70(s,3H,CH_3_), 3.83(s,3H,CH_3_), 6.85 (d,2H,* J* = 7.8 Hz), 6.93(t,1H,* J* = 8.4 Hz), 7.04(t,1H,* J* = 7.2 Hz), 7.14 (d,1H,* J* = 7.8 Hz), 7.21–7.24 (m,2H), 7.35(d,2H,* J* = 8.4 Hz), 7.40 (d,1H,* J* = 7.8 Hz), 8.08 (d,1H,* J* = 7.8 Hz),8.14 (s,1H),8.89(s,1H),9.16 (s,1H), 11.83(s,1H); ^13^C- NMR ppm;55.2,56.0,114.1,115.5, 118.7, 118.9, 120.0, 120.4, 121.5, 122.6, 123.0, 127.0, 132.7, 133.5, 138.8, 148.0, 149.6, 152.2, 154.6, 162.5; C_22_H_21_N_3_O_4_(391.43) m/z (%): 492.16[M + H] + (100).

##### (E)-1-(4-chlorophenyl)-3-(2-((2-hydroxy-3-methoxybenzylidene) amino) phenyl) urea (3q)

Yellow solid; Yield: 62%, m.p: 218–220 °C; IR ʋ max (cm^−1^): 3300 (NH), 1613 (C=N), 1649(C=O), ^1^H-NMR (DMSO-*d*^6^) δ ppm;(s,3H,CH_3_) 6.91(t,1H,* J* = 7.8 Hz), 7.01 (d,1H,* J* = 7.8 Hz), 7.09 (t,1H,* J* = 7.2 Hz), 7.14 (d,1H,* J* = 7.8 Hz), 7.22–7.30 (m,4H), 7.39 (d,1H,* J* = 7.2 Hz), 7.73 (s,1H), 8.04 (d,1H,* J* = 7.8 Hz),8.27 (s,1H),8.89 (s,1H),9.54 (s,1H), 11.91(s,1H); ^13^C- NMR ppm;14.7,64.1, 116.6, 116.7, 117.6, 118.8, 118.9, 120.3, 120.5, 121.5, 123.2, 123.3, 127.0, 130.4, 132.8, 133.2, 139.2, 141.2, 147.9, 152.2, 163.1;C_21_H_18_ClN_3_O_3_ (395.84) m/z (%): 396.10[M + H] + (100).

##### (E)-1-(2-((2-hydroxy-3-methoxybenzylidene) amino) phenyl)-3-(o-tolyl) urea (3r)

Cream Yellow solid; Yield: 55%, m.p: 210–212 °C; IR ʋ max (cm^−1^): 3300 (NH), 1613 (C=N), 1649(C=O), ^1^H-NMR (DMSO-*d*^6^) δ ppm; 2.18(s,3H,CH_3_), 3.78(s,3H,CH_3_), 6.81(t,1H,* J* = 7.8 Hz), 6.86–6.90 (m,2H), 6.97–6.91 (m,2H), 7.08–7.11 (m,2H), 7.24–7.26 (m,1H), 7.54 (d,1H,* J* = 7.8 Hz), 7.62 (s,1H), 8.28 (d,1H,* J* = 7.8 Hz),8.43(s,1H), 12.78(s,1H); ^13^C- NMR ppm;17.9,55.8,114.7, 118.6, 119.0, 119.2, 120.4, 123.0, 123.8, 124.6, 125.2, 126.8, 127.9, 130.7, 132.8, 135.8, 138.3, 148.0, 150.3, 152.3, 164.1; C_22_H_21_N_3_O_3_(375.43) m/z (%): 376.16[M + H] + (100).

##### (E)-1-(2-((2-hydroxy-3-methoxybenzylidene) amino) phenyl)-3-(m-tolyl) urea (3s)

Yellow solid; Yield: 50%, m.p: 201–203 °C; IR ʋ max (cm^−1^): 3300 (NH), 1613 (C=N), 1649(C=O), ^1^H-NMR (CDCl_3_) δ ppm;2.22 (s,3H, CH_3_), 3.74 (s,3H, CH_3_), 6.77–6.82 (m,2H), 6.86(d,1H,* J* = 7.8 Hz), 6.89(d,1H,* J* = 10.2 Hz), 6.97(d,1H,* J* = 7.8 Hz), 7.02(t,1H,* J* = 7.8 Hz), 7.04–7.07 (m,2H), 7.23–7.26 (m,2H), 7.63(s,1H),7.79(s,1H), 8.31(d,1H,* J* = 7.8 Hz),8.46(s,1H),13.051(s,1H); ^13^C- NMR δppm; 21.74, 55.7, 114.7, 116.9, 118.6, 119.0, 119.3, 120.3, 120.6, 122.8, 123.9, 127.9, 128.7, 133.0, 138.1, 138.4, 138.8, 148.0, 150.2, 152.8, 164.2; C_22_H_21_N_3_O_3_(375.43) m/z (%): 376.16[M + H] + (100).

##### (E)-1-(2-((2-hydroxy-3-methoxybenzylidene) amino) phenyl)-3-(p-tolyl) urea (3t)

Yellow solid; Yield: 50%, m.p: 218–200 °C; IR ʋ max (cm^−1^): 3300 (NH), 1613 (C=N), 1649(C=O), ^1^H-NMR (CDCl_3_) δ ppm;2.21 (s,3H, CH_3_), 3.82 (s,3H, CH_3_), 6.84(t,1H,* J* = 7.8 Hz), 6.92–7.01 (m,6H), 7.20–7.24 (m,3H), 7.63(s,1H),8.01(s,1H), 8.25(d,1H,* J* = 8.4 Hz),8.49(s,1H),12.81(s,1H); ^13^C-NMR δppm; 20.7, 56.0, 114.9, 118.6, 119.0, 119.4, 120.1, 120.5, 122.8, 124.0, 127.8, 129.5, 132.5, 133.1, 136.2, 138.4, 148.2, 150.4, 153.1, 164.2; C_22_H_21_N_3_O_3_(375.43) m/z (%): 376.16[M + H] + (100).

##### (E)-1-(2-((2-hydroxy-3-methoxybenzylidene) amino) phenyl)-3-phenylurea (3u)

Yellow solid; Yield: 50%, m.p: 188–190 °C; IR ʋ max (cm^−1^): 3300 (NH), 1613 (C=N), 1649(C=O), ^1^H-NMR (CDCl_3_) δ ppm;3.82(s,3H,CH_3_) 6.84(t,1H,* J* = 7.8 Hz), 6.92–7.02 (m,5H), 7.19–7.24 (m,3H), 7.37 (d,1H,* J* = 7.8 Hz), 7.73 (s,1H), 8.25 (d,1H,* J* = 8.4 Hz), 8.28 (s,1H),8.51 (s,1H), 12.89(s,1H); ^13^C- NMR ppm; 56.0, 115.0,118.7,119.1,119.5,120.7, ,122.7,124.0,127.8,128.9, 133.0,138.5,139.2, 148.2,150.4,152.9,164.2;C_21_H_19_N_3_O_3_ (361.40) m/z (%): 362.15[M + H] + (100).

##### (E)-1-(4-acetylphenyl)-3-(2-((2-hydroxy-3-methoxybenzylidene) amino) phenyl) urea (3v)

Yellow solid; Yield: 74%, m.p: 200–212 °C; IR ʋ max (cm^−1^): 3300 (NH), 1613 (C=N), 1649(C=O), ^1^H-NMR (DMSO-*d*^6^) δ ppm; (s,3H,CH_3_) 6.83(t,1H,* J* = 7.8 Hz), 6.88(d,1H,* J* = 7.8 Hz), 6.92 (d,1H,* J* = 7.2 Hz), 7.01 (d,1H,* J* = 7.8 Hz), 7.06 (t,1H,* J* = 7.8 Hz), 7.27 (t,1H,* J* = 7.8 Hz), 7.45 (d,1H,* J* = 8.4 Hz), 7.79 (d,1H,* J* = 8.4 Hz), 7.86 (s,1H), 8.27 (d,1H,* J* = 7.8 Hz),8.50 (s,1H),8.57 (s,1H), 13.13(s,1H); ^13^C- NMR ppm;26.3,55.8,114.8, 117.8, 118.7, 119.3, 120.7, 120.5, 123.4, 124.0, 128.0, 129.8, 131.2, 132.6,138.2,139.4,144.0,148.1,152.3,164.3,197.1; C_23_H_21_N_3_O_4_(403.44) m/z (%): 404.16[M + H] + (100).

##### (E)-1-(4-chlorophenyl)-3-(2-((4-hydroxy-3-methoxybenzylidene) amino) phenyl) urea (3w)

Greenish offwhite solid; Yield: 52%, m.p:219- 221 °C; IR ʋ max (cm^−1^): 3300 (NH), 1613 (C=N), 1649(C=O), ^1^H-NMR (DMSO-*d*^6^) δ ppm;3.9(s,3H,CH_3_) , 6.92(d,1H,* J* = 7.8 Hz), 6.99(t,1H,* J* = 7.8 Hz), 7.16 (t,1H,* J* = 7.2 Hz), 7.25 (d,1H,* J* = 7.8 Hz), 7.31 (d,2H,* J* = 7.8 Hz), 7.49 (d,3H,* J* = 7.2 Hz), 7.70 (d,1H,* J* = 7.2 Hz), 8.22 (d,1H,* J* = 8.4 Hz),8.46(s,1H),8.60 (s,1H),9.74 (s,1H), 9.80(s,1H); ^13^C- NMR ppm; 55.8,111.8, 115.5,117.1,118.3,119.7,122.0, 124.5, 126.4,127.9,128.7, 134.1,138.7,138.8,148.0,150.6,152.1,159.6; C_21_H_18_ClN_3_O_3_ (395.84) m/z (%): 396.10[M + H] + (100).

##### (E)-1-(2-((4-hydroxy-3-methoxybenzylidene) amino) phenyl)-3-(o-tolyl) urea (3x)

Greenish offwhite; Yield: 54%, m.p: 200–202 °C; IR ʋ max (cm^−1^): 3300 (NH), 1613 (C=N), 1649(C=O), ^1^H-NMR (DMSO-*d*^6^) δ ppm; 2.24(s,3H,CH_3_), 3.33(s,3H,CH_3_), 6.92–6.99 (m,3H), 7.13–7.18 (m,3H), 7.26 (dd,1H,* J* = 7.8,0.6 Hz) , 7.42 (dd,1H,* J* = 6.6,1.8 Hz) , 7.64 (d,1H* J* = 7.8 Hz), 8.18 (dd,1H,* J* = 7.2,1.2 Hz) ,8.61(s,1H), 8.64(s,1H),8.75(s,1H), 9.78(s,1H); ^13^C- NMR ppm;18.1, 55.7, 111.7, 115.4, 117.0, 118.9, 121.9, 123.1, 123.5, 124.5, 126.1, 126.4, 128.0, 129.5, 130.3, 134.4, 137.2, 138.7, 148.0, 150.5, 152.8 ,159.2; C_22_H_21_N_3_O_3_ (375.43) m/z (%): 376.16[M + H] + (100).

##### (E)-1-(2-((4-hydroxy-3-methoxybenzylidene) amino) phenyl)-3-(p-tolyl) urea (3y)

Greenish offwhite; Yield: 40%, m.p: 209–211 °C; IR ʋ max (cm^−1^): 3300 (NH), 1613 (C=N), 1649(C=O), ^1^H-NMR (CDCl_3_) δ ppm; 3.32(s,3H,CH_3_), 3.89(s,3H,CH_3_), 6.92(d,1H* J* = 7.8 Hz), 6.97 (td,1H,* J* = 7.8,1.2 Hz), 7.08 (d,2H* J* = 8.4 Hz), 7.15 (td,1H,* J* = 8.4,1.2 Hz) , 7.23 (dd,1H,* J* = 8.4,1.2 Hz), 7.34 (d,2H* J* = 9 Hz), 7.49(dd,1H,* J* = 8.4,1.8 Hz) , 7.69 (d,1H* J* = 1.8 Hz), 8.23(dd,1H,* J* = 8.8,1.2 Hz) ,8.39(s,1H), 8.59(s,1H),9.47(s,1H), 9.79(s,1H); 13CNMRδppm; 20.4, 55.8, 111.9, 115.5, 117.1, 118.2, 118.5, 121.8, 124.5, 126.1, 126.4, 127.9, 129.2, 130.7, 134.4, 137.2, 138.6, 148.0, 150.6, 152.3, 159.4;C_22_H_21_N_3_O_3_(375.43) m/z (%): 376.16[M + H] + (100).

#### α-glucosidase inhibition assay

The inhibition of *α*-Glucosidase (E.C.3.2.1.20) enzyme was performed by using assay 0.05 M phosphate buffer (pH 6.8) at 37 °C^27^. At 37 °C for 15 min, the enzyme (2 Units/2 mL) was incubated in phosphate-buffer with various concentrations of the tested substances dissolved in DMSO. Afterwards, the substrate (0.7 mM, p-nitrophenyl-* α*-D-glucopyranoside) was added, and the variation in absorbance at 400 nm was measured through spectrophotometer (xMarkTM Microplate Spectrophotometer, BIO-RAD) for 30 min. In the control, the tested compounds were replaced with DMSO-d6 (7.5 percent final). As a standard inhibitor, acarbose was utilized.

### Statistical analysis

SoftMax Pro suite and Excel were used to analyse the obtained results for biological activity. Percent inhibition was calculated using the given formula (Eq. 1).

1 $$\begin{array}{*{20}c} {\% Inhibition = 100 - \left( {\frac{{O.D_{test \;compound} }}{{O.D_{control} }}} \right) \times 100} \\ \end{array}$$

EZ-FIT (Perrella Scientific, Inc., USA) was used for IC_50_ calculations of all tested samples. To overcome on the expected errors, all experiments were performed in triplicate, and variations in the results are reported in Standard Error of Mean values (SEM) (Eq. 2).

2 $$\begin{array}{*{20}c} {SE = \frac{\sigma }{\sqrt n }} \\ \end{array} .$$

### Molecular docking

In the molecular docking study, the X-ray crystal structure of isomaltase from *Saccharomyces cerevisiae* was used in complex with α-D-glucopyranose (PDB code: 3A4A, resolution: 1.60 Å)^28^. The docking experiment was carried out on Molecular Operating Environment (MOE version 2020.0901)^29^. Previously, we have tested the docking performance of MOE through re-docking protocol and MOE showed good efficiency^5,7,29,30^. In this work, the protein file was prepared for docking by QuickPrep module of MOE which add missing hydrogens on each residue of protein to fulfil their valency and calculates partial charges (via Amber10: EHT force field). While the structures of compounds were drawn by ChemDraw and imported into MOE database where all the structures were converted into three-dimensional (3D)-format by MOE-WASH module which all hydrogen atoms and partial charges on all the compounds and minimize the structure of each ligand with RMS gradient of 0.1RMS kcal/mol/Å. After the preparation of protein and ligand files, docking was performed with Triangle Matcher docking algorithm and London dG scoring function. When the docking was finished, conformational sampling was performed to select the best docked conformation of each ligand based on good docking score and good binding interaction.

### ADMET calculation

The pharmacokinetic profile and drug-likeness and medicinal chemistry properties of compounds were predicted through SwissADME server^31^. Each compound was uploaded on the server in SMILE format to predict their ADMET properties.

## Conclusion

Type II diabetes is a serious health issue with high glycemic effect and can be controlled by α-glucosidase inhibitors as therapeutic approach. In search of non-sugar based *α*-glucosidase inhibitors, a new series of Schiff bases of 1,3-dipheny urea (**3a–y**) were designed and synthesized. All the synthesized chemical analogues were scrutinized for in vitro* α*-glucosidase enzyme inhibitory potential, which clearly demonstrated their role in T2DM. Most of the compounds displayed excellent potency with lower IC_50_ values. The structure–activity relationship of this series showed that diversity in R_1_ and R_2_-groups displayed a key role in the inhibitory capability of *α*-glucosidase. The docking studies showed that all compounds are well fitted in the active site of α-glucosidase, where Glu277 and Asn350 are mainly stabilize the binding of these compounds. Moreover, predicted ADMET profile reflect that the synthesized molecules are good option of druglike candidates. Further studies on the structural optimization of these derivatives are underway in our laboratory.

## Supplementary Information

Supplementary Information.

## Data Availability

All data generated or analyzed during this study are included in this published article [and its supplementary information files].
