# Supplementary material for: Synthesis of new diphenyl urea-clubbed imine analogs and its Implications in diabetic management through in vitro and in silico approaches
Source: Sci Rep. 2023 Feb 1;13:1877. doi: 10.1038/s41598-023-28828-1 (PMC9892044; doi:10.1038/s41598-023-28828-1)
Supplement: Supplementary file 1 — Supplementary Information. [file 41598_2023_28828_MOESM1_ESM.docx]

**Supporting information**

# **Synthesis of new diphenyl urea-clubbed imine analogs and its Implications in diabetic management through *in vitro* and *in silico* approaches**

Anam Rubbab Pasha^a,b^, Ajmal Khan^b^, Saeed Ullah^b,c^, Sobia Ahsan Halim^b^, Javid Hussain^b^, Muhammad Khalid^d,e^, Muhammad Moazzam Naseer^f^, Attalla F. El‑kott^g,h^, Sally Negm^i,j^, Ahmed Al-Harrasi^b^* and Zahid Shafiq ^a,k^*

*^a^Institute of Chemical Sciences, Bahauddin Zakariya University, Multan-60800, Pakistan*

*^b^Natural and Medical Sciences Research Center, University of Nizwa, Birkat-ul-Mouz 616, Nizwa, Sultanate of Oman*

*^c^H. E. J. Research Institute of Chemistry, International Center for Chemical and Biological Sciences, University of Karachi, Karachi-75270, Pakistan*

*^d^Department of Chemistry, Khwaja Fareed University of Engineering & Information Technology, Rahim Yar Khan, 64200, Pakistan*

*^e^Centre for Theoretical and Computational Research, Khwaja Fareed University of Engineering & Information Technology, Rahim Yar Khan, 64200, Pakistan*

*^f^Department of Chemistry, Quaid-i- Azam University, Islamabad, 45320, Pakistan*

*^g^Department of Biology, College of Science, King Khalid University, Abha 61421, Saudi Arabia*

*^h^Department of Zoology, College of Science, Damanhour University, Damanhour 22511, Egypt*

*^i^Department of Life Sciences, College of Science and Art Mahyel Aseer, King Khalid University, Abha 62529, Saudi Arabia*

*^j^Unit of Food Bacteriology, Central Laboratory of Food Hygiene, Ministry of Health, Branch in Zagazig, Zagazig 44511, Egypt*

*^k^Department of Pharmaceutical & Medicinal Chemistry, An der Immenburg 4, D-53121 Bonn, Germany*

*Corresponding author:

Zahid Shafiq: e-mail: [zahidshafiq@bzu.edu.pk](mailto:zahidshafiq@bzu.edu.pk), Tel. +92-3006559811.

Ahmed Al-Harrasi: e-mail: [aharrasi@unizwa.edu.om](mailto:aharrasi@unizwa.edu.om), Tel. +96825446328.

**(3a) (E)-1-(3-chlorophenyl)-3-(2-((3-ethoxy-2-hydroxybenzylidene)amino)phenyl)urea**

C_22_H_20_ClN_3_O_3_ (409.12)
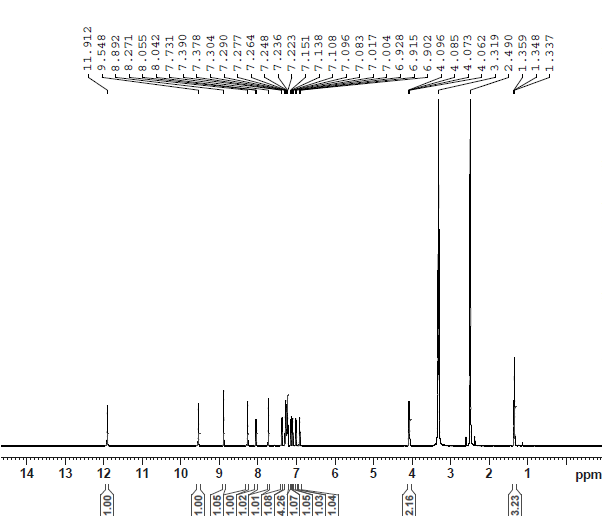


**(3a) H^1^NMR**


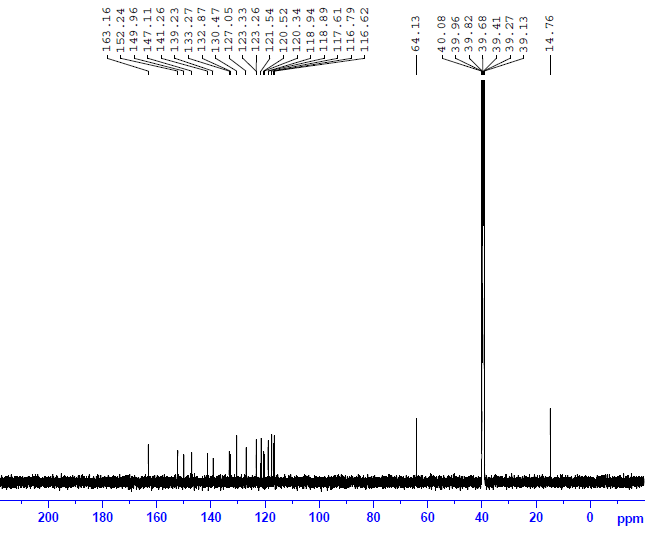


**(3a) C^13^NMR**


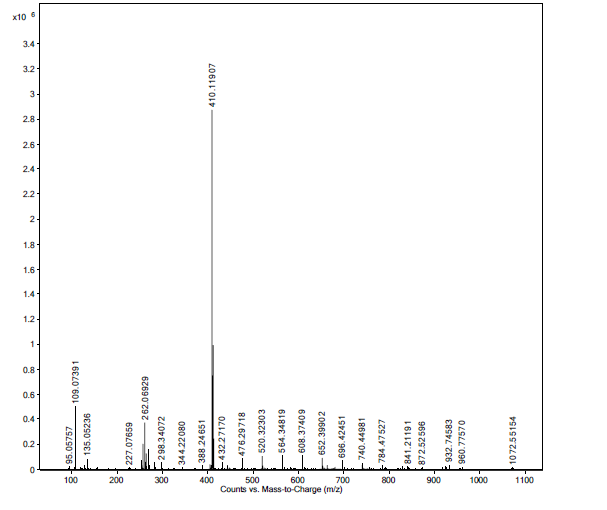


**(3a) Mass spectrum**

**(3b) (E)-1-(2-((3-ethoxy-2-hydroxybenzylidene)amino)phenyl)-3-(4-fluorophenyl)urea**

C_22_H_20_FN_3_O_3_ (393.42)


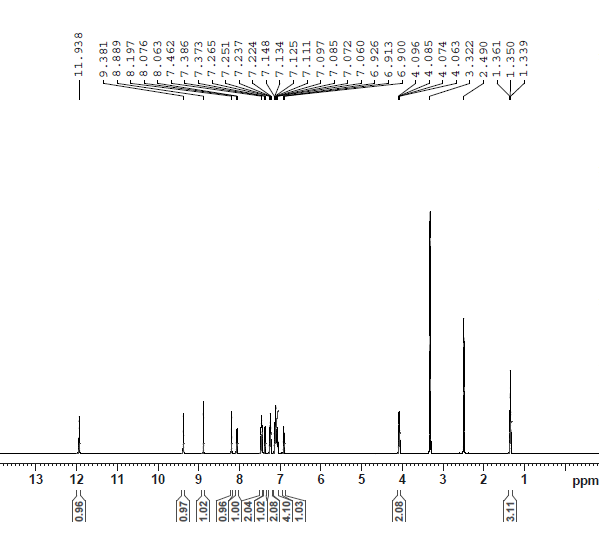


**(3b) H^1^NMR**


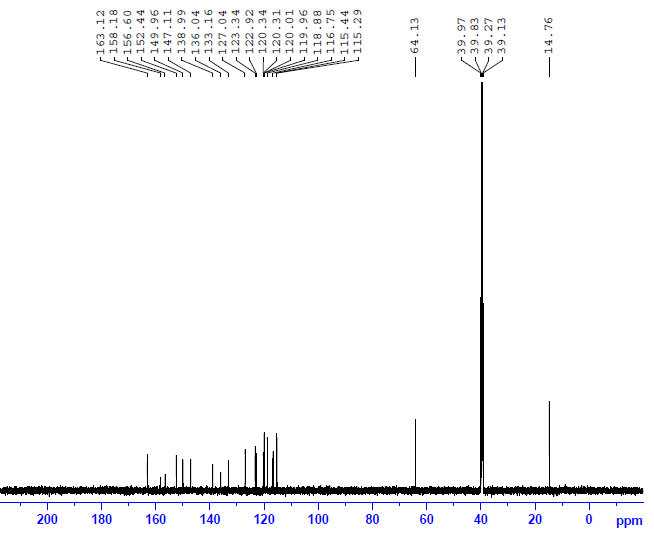


**(3b) C^13^NMR**


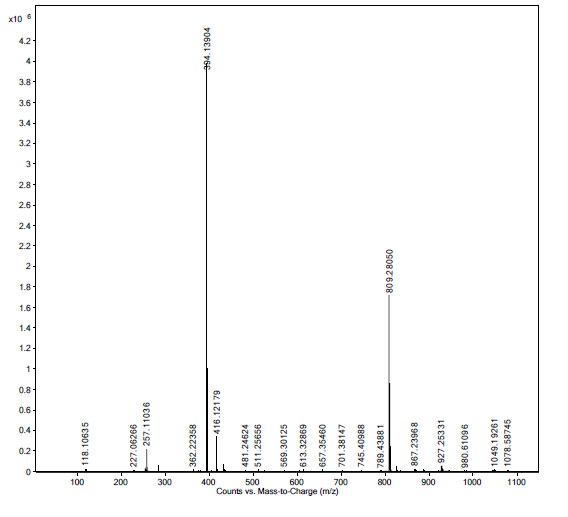


**(3b) Mass spectrum**

**(3c)** (E)-1-(2-((3-ethoxy-2-hydroxybenzylidene)amino)phenyl)-3-(3-fluorophenyl)urea

C_22_H_20_FN_3_O_3_ (393.42)


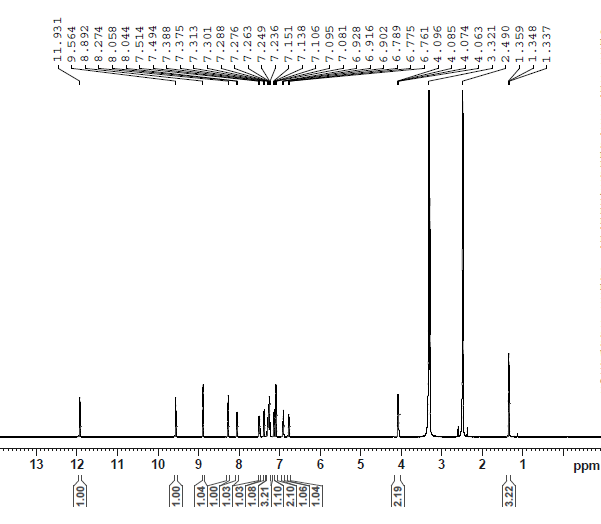


**(3c) H^1^NMR**


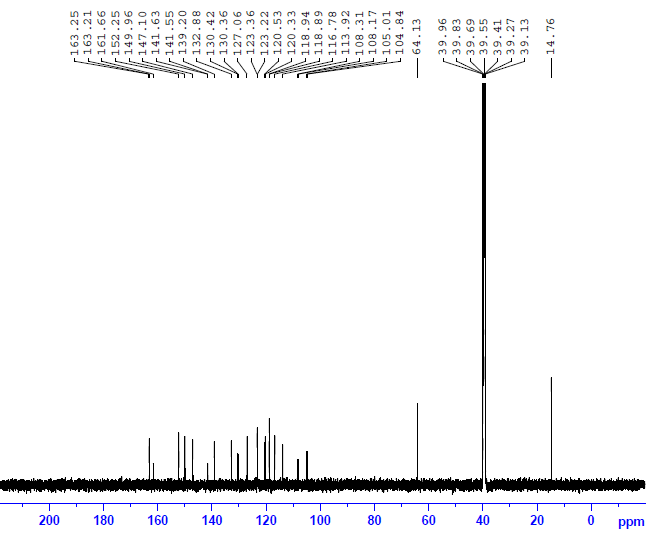


**(3c) C^13^NMR**


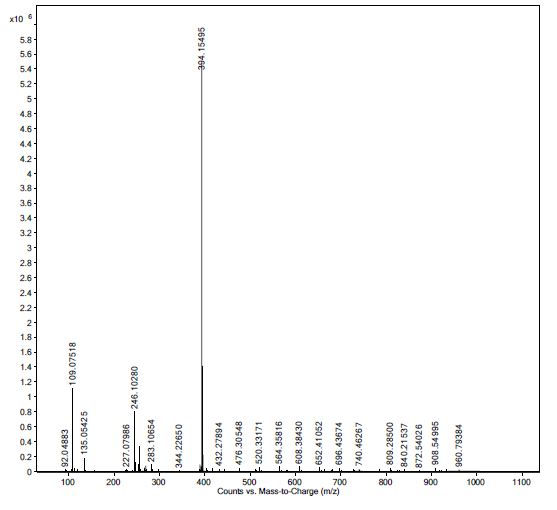


**(3c) Mass spectrum**

**(3d)** (E)-1-(2-((3-ethoxy-2-hydroxybenzylidene)amino)phenyl)-3-(naphthalen-2-yl)urea

C_26_H_23_N_3_O_3_ (425.49)


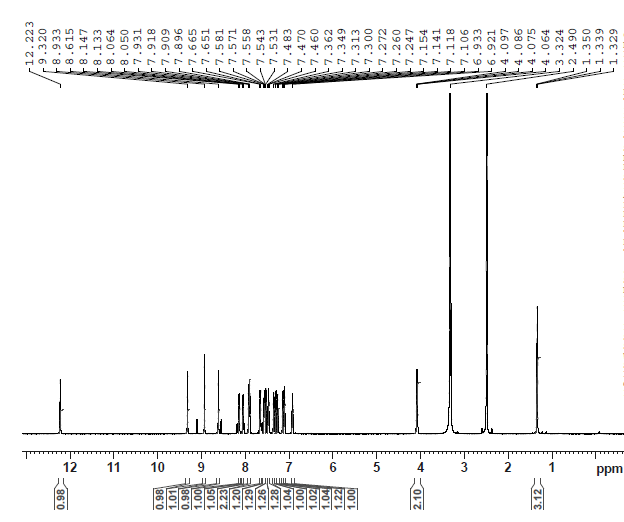


**(3d) H^1^NMR**


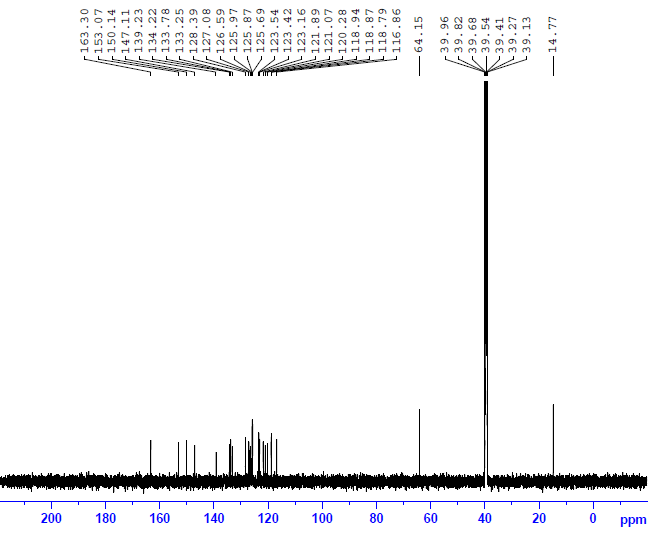


**(3d) C^13^NMR**


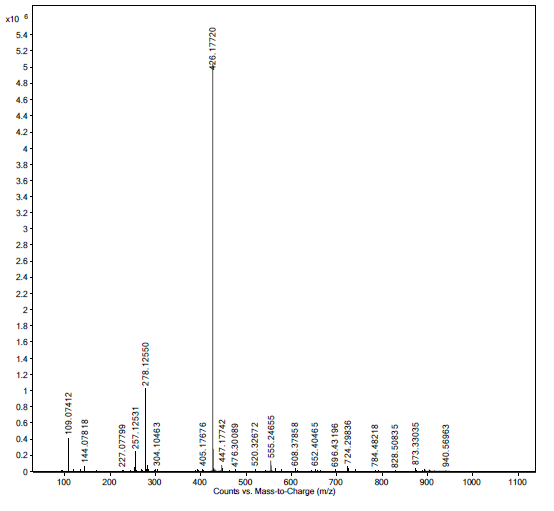


**(3d) Mass spectrum**

**(3e)** (E)-1-(2-((3-ethoxy-2-hydroxybenzylidene)amino)phenyl)-3-(4-methoxyphenyl)urea

C_23_H_23_N_3_O_4_ (405.45)


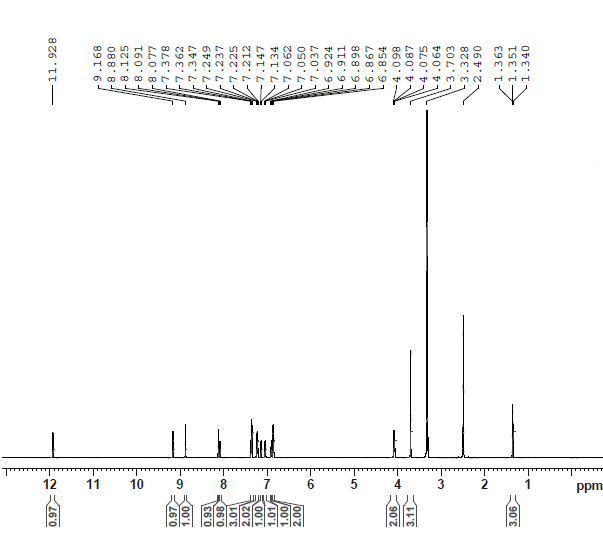


**(3e) H^1^NMR**


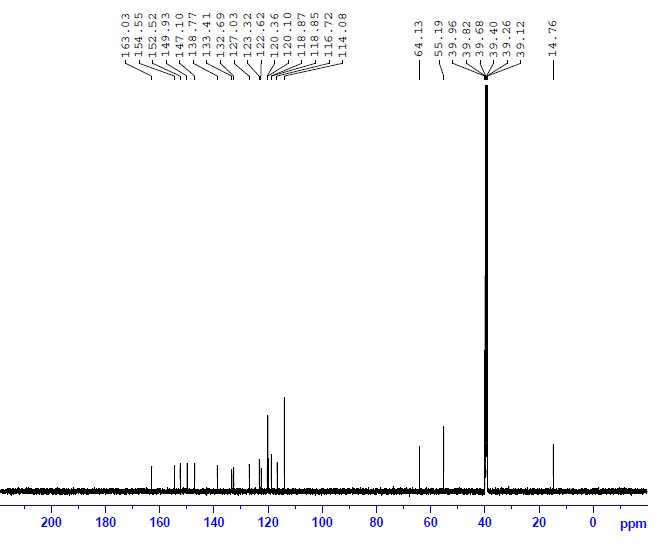


**(3e) C^13^NMR**


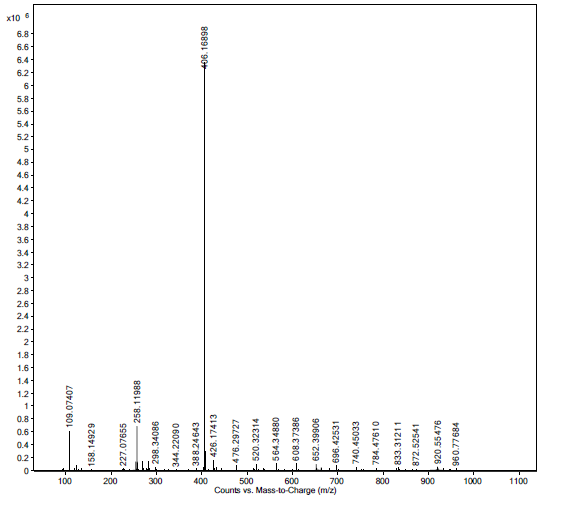


**(3e) Mass spectrum**

**(3f)** (E)-1-(4-chlorophenyl)-3-(2-((3-ethoxy-2-hydroxybenzylidene) amino) phenyl) urea

C_22_H_20_ClN_3_O_3_ (409.12)
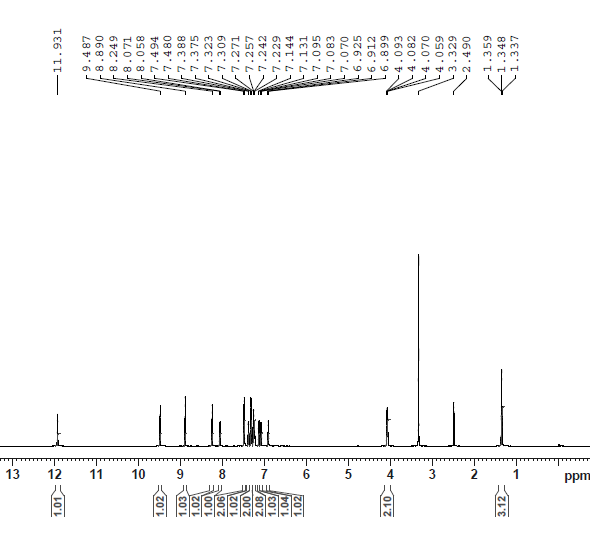


**(3f) H^1^NMR**


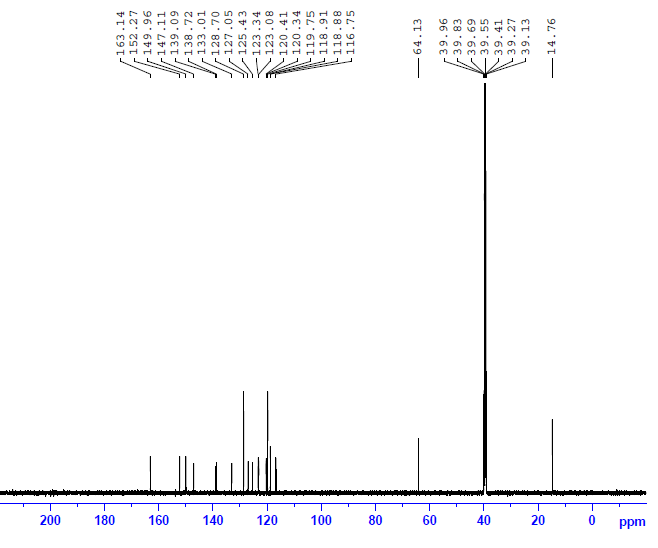


**(3f) C^13^NMR**


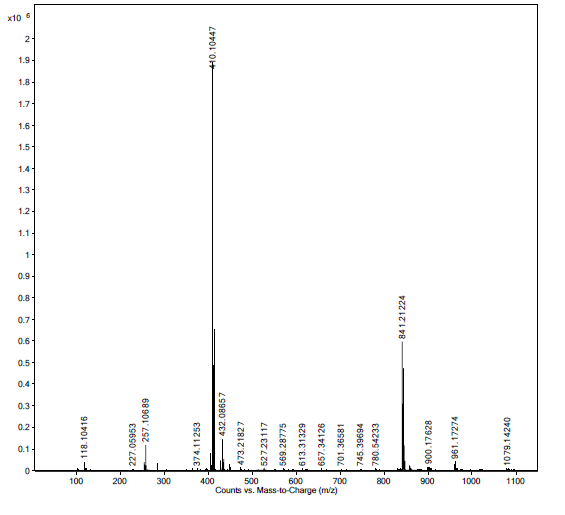


**(3f) Mass spectrum**

**(3g)** (E)-1-(2-((3-ethoxy-2-hydroxybenzylidene)amino)phenyl)-3-(o-tolyl)urea

C_23_H_23_N_3_O_3_ (389.46)
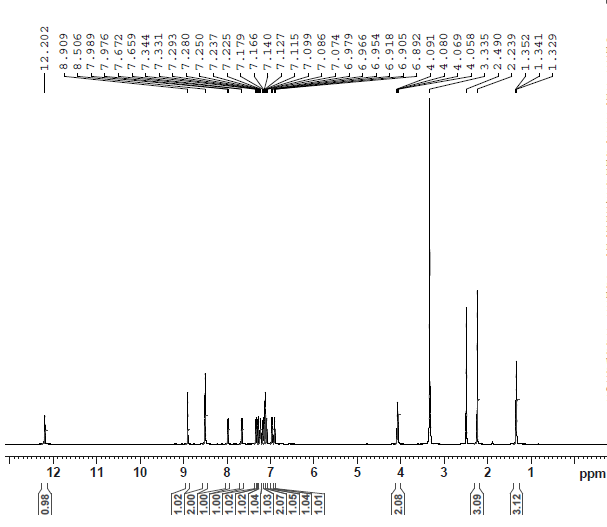


**(3g) H^1^NMR**


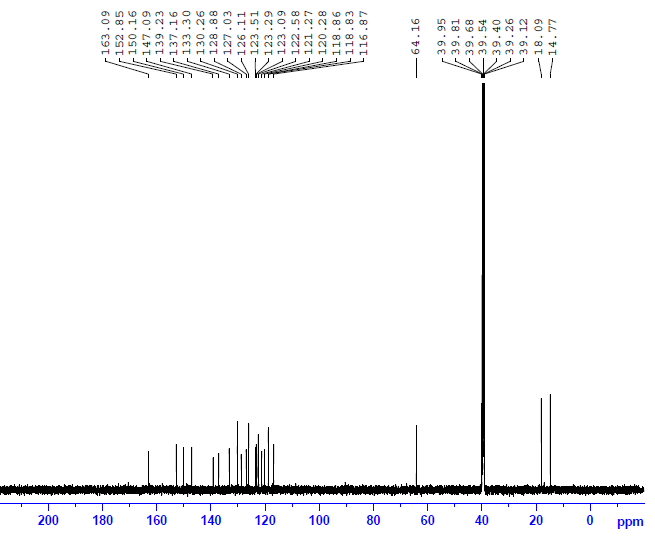


**(3g) C^13^NMR**


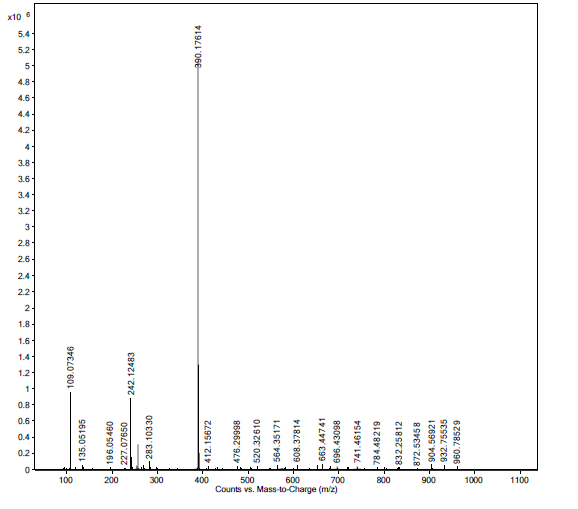


**(3g) Mass Spectrum**

**(3h)** (E)-1-(2-((3-ethoxy-2-hydroxybenzylidene)amino)phenyl)-3-(m-tolyl)urea

C_23_H_23_N_3_O_3_ (389.46)
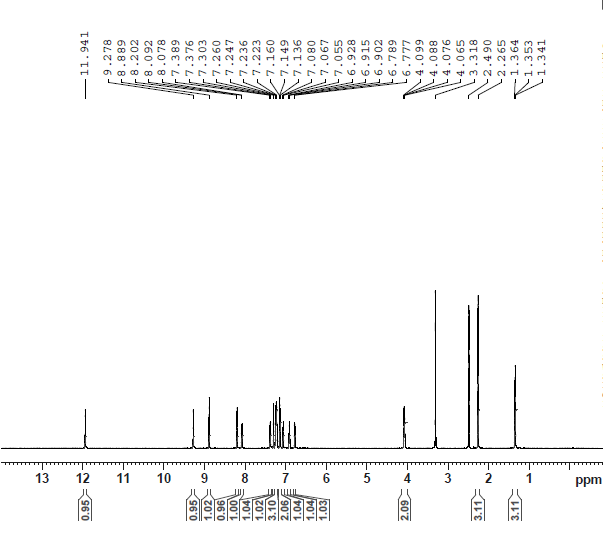


**(3h) H^1^NMR**


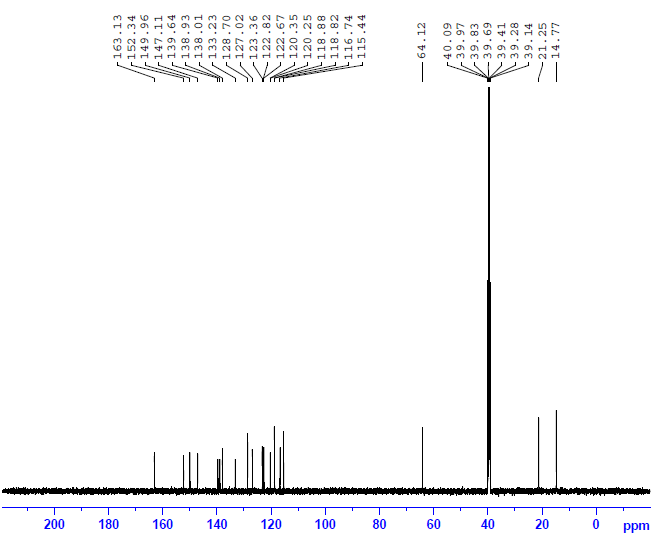


**(3h) C^13^NMR**


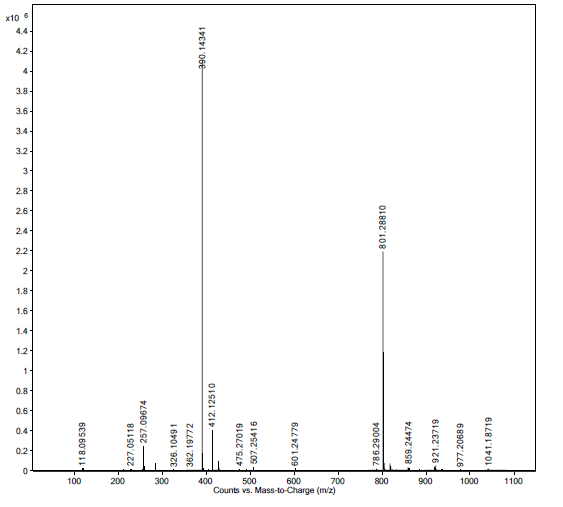


**(3h) Mass spectrum**

**(3i)** (E)-1-(2-((3-ethoxy-2-hydroxybenzylidene)amino)phenyl)-3-(p-tolyl)urea

C_23_H_23_N_3_O_3_ (389.46)


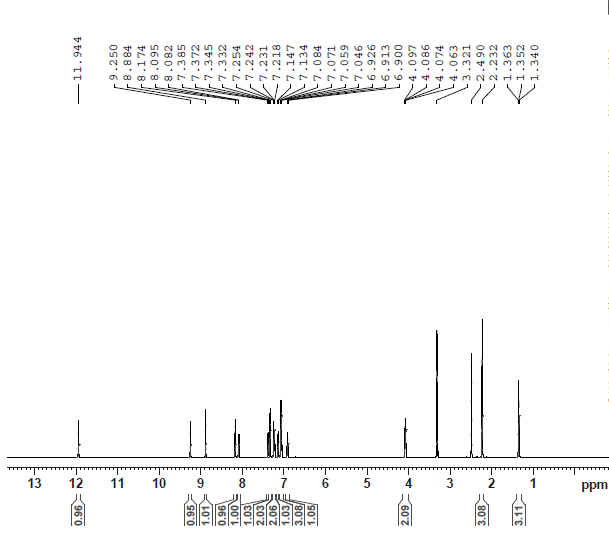


**(3i) H^1^NMR**


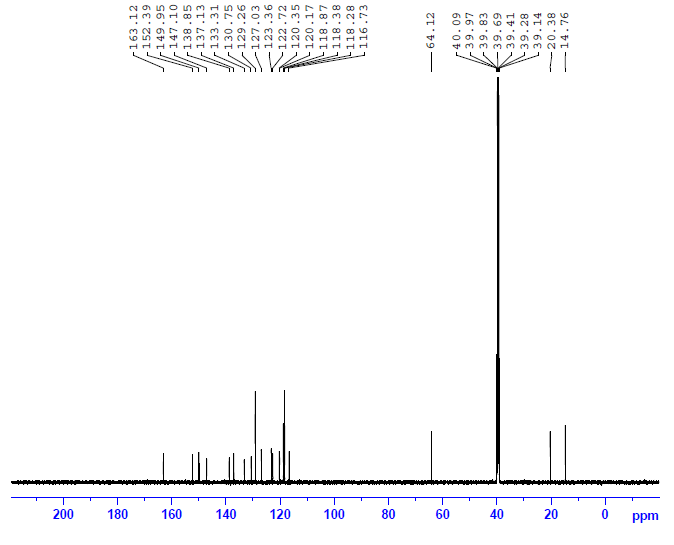


**(3i) C^13^NMR**


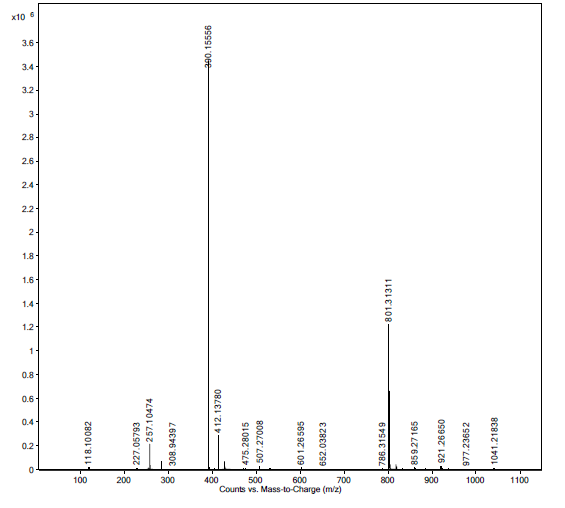


**(3i) Mass spectrum**

**(3j)** (E)-1-(2-((3-ethoxy-2-hydroxybenzylidene)amino)phenyl)-3-phenylurea

C_22_H_21_N_3_O_3_ (375.16)


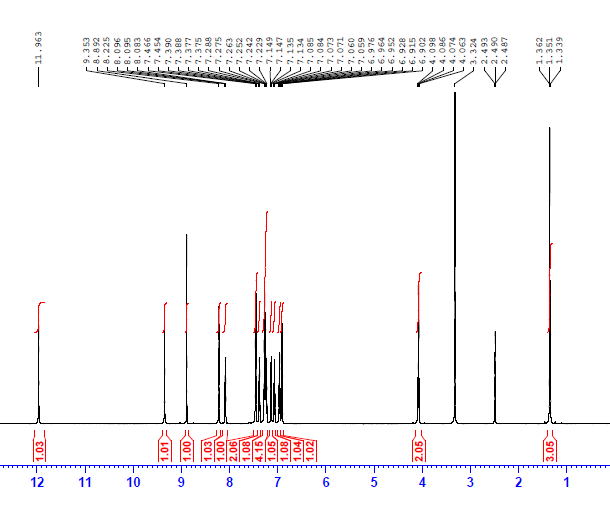


**(3j) H^1^NMR**


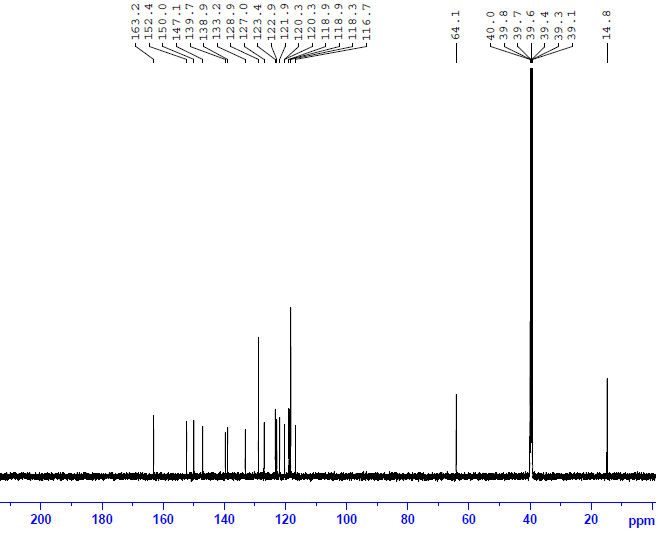


**(3j) C^13^NMR**

**(3k)** (E)-1-(4-acetylphenyl)-3-(2-((3-ethoxy-2-hydroxybenzylidene)amino)phenyl)urea

C_24_H_23_N_3_O_4_ (417.47)


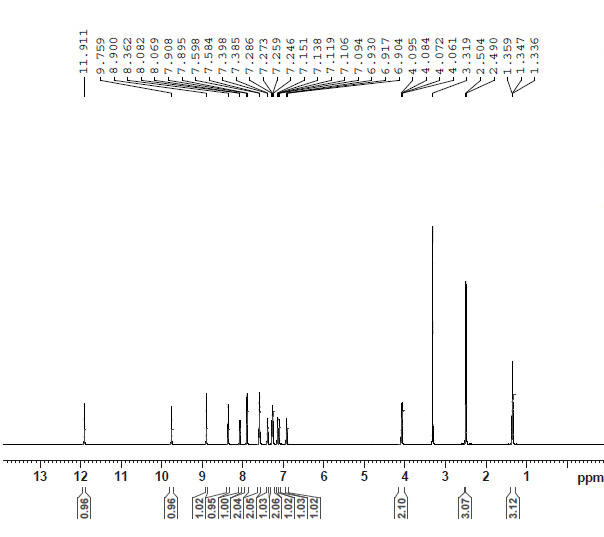


**(3k) H^1^NMR**


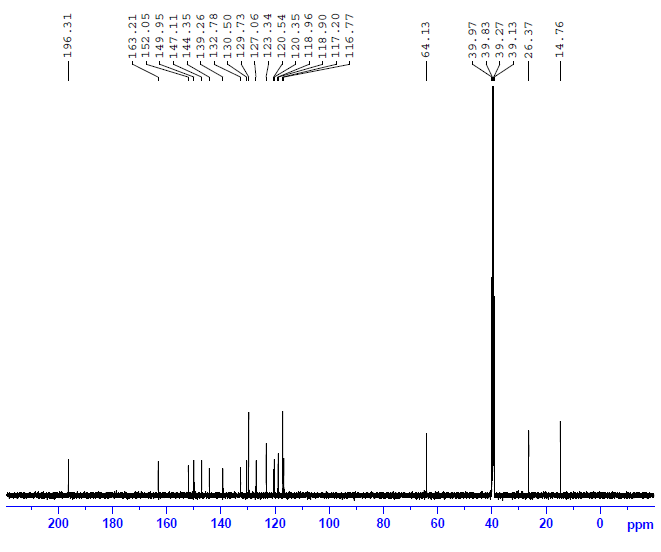


**(3k) C^13^NMR**


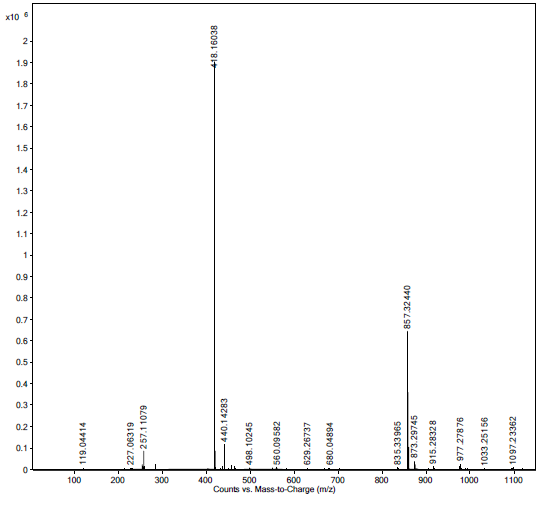


**(3k) Mass spectrum**

**(3l)** (E)-1-(3-chlorophenyl)-3-(2-((2-hydroxy-3-methoxybenzylidene)amino)phenyl)urea

C_21_H_18_ClN_3_O_3_ (395.84)


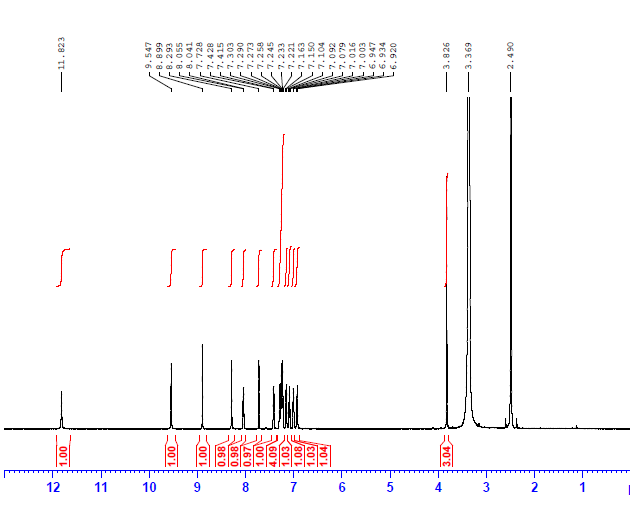


**(3l) H^1^NMR**


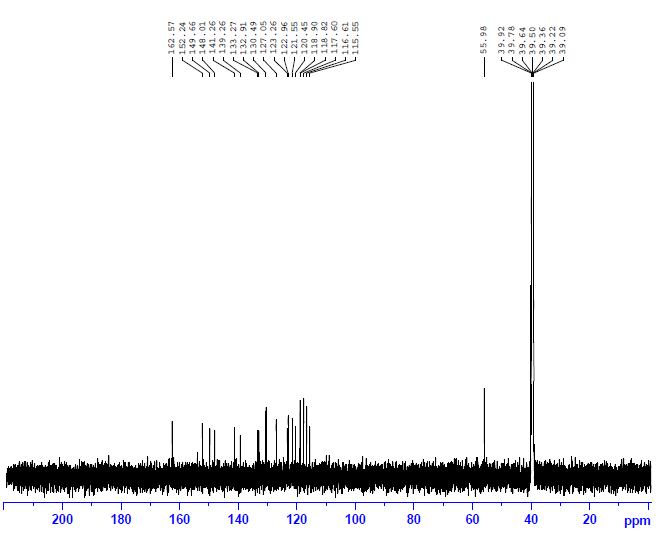


**(3l) C^13^NMR**


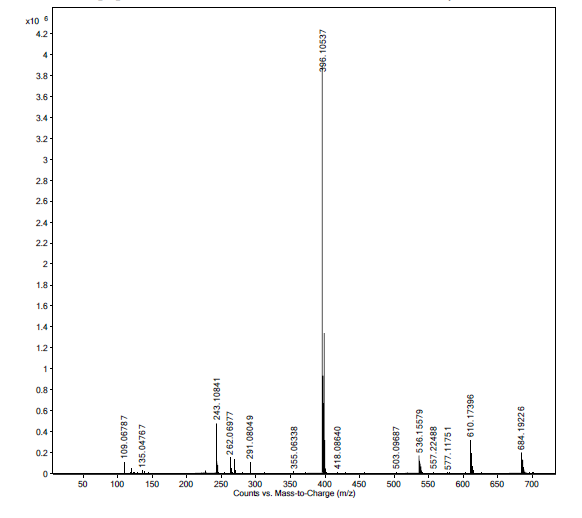


**(3l) Mass spectrum**

**(3m)** (E)-1-(4-fluorophenyl)-3-(2-((2-hydroxy-3-methoxybenzylidene)amino)phenyl)urea

C_21_H_18_FN_3_O_3_ (379.39)


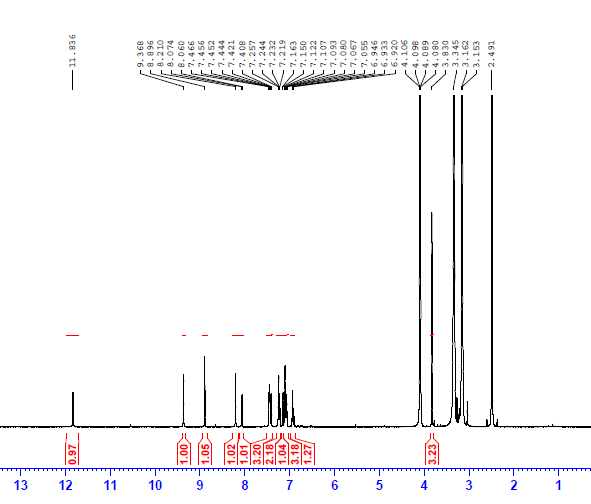


**(3m) H^1^NMR**


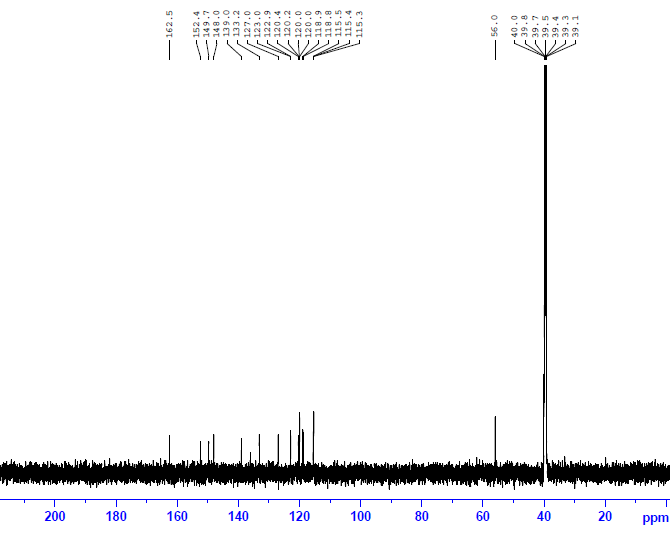


**(3m) C^13^NMR**


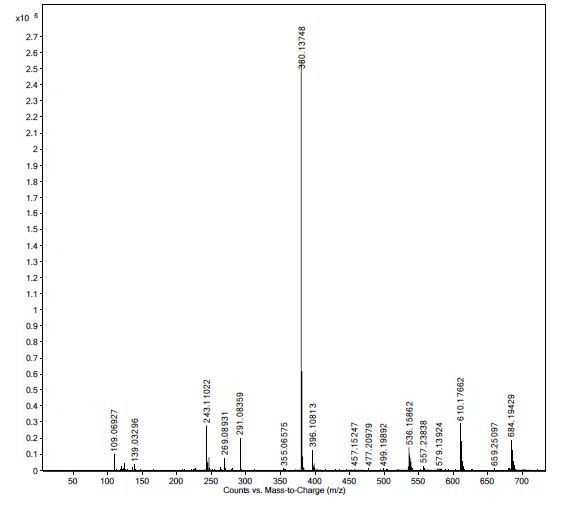


**(3m) Mass spectrum**

**(3n)** (E)-1-(3-fluorophenyl)-3-(2-((2-hydroxy-3-methoxybenzylidene)amino)phenyl)urea

C_21_H_18_FN_3_O_3_ (379.39)


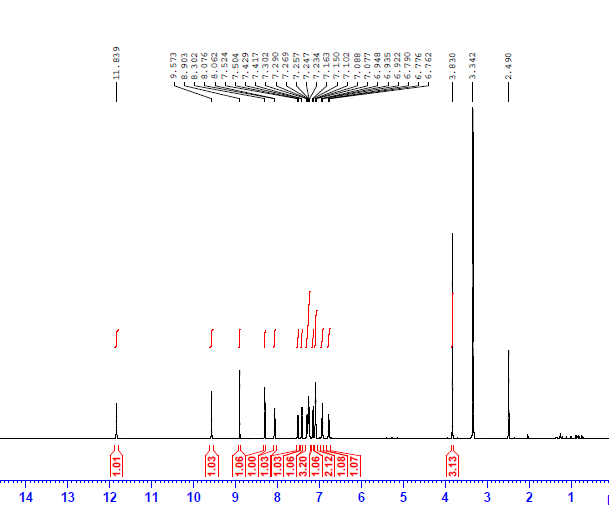


**(3n) H^1^NMR**


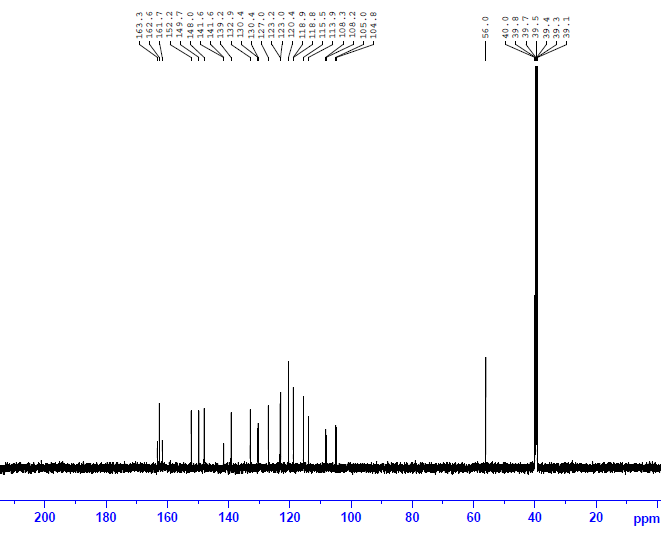


**(3n) C^13^NMR**


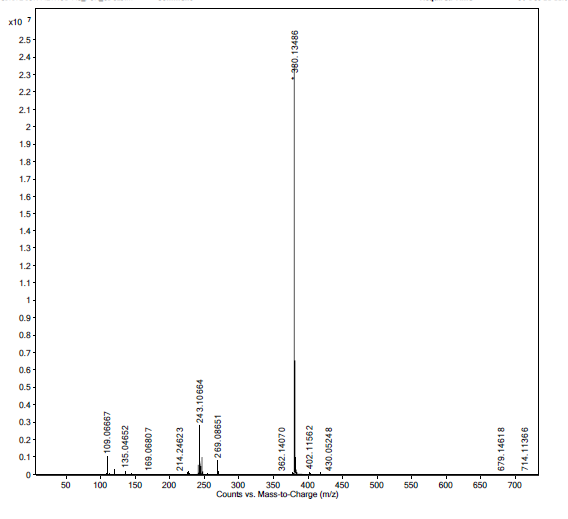


**(3n) Mass spectrum**

**(3o)** (E)-1-(2-((2-hydroxy-3-methoxybenzylidene)amino)phenyl)-3-(naphthalen-2-yl)urea

C_25_H_21_N_3_O_3_ (411.46)


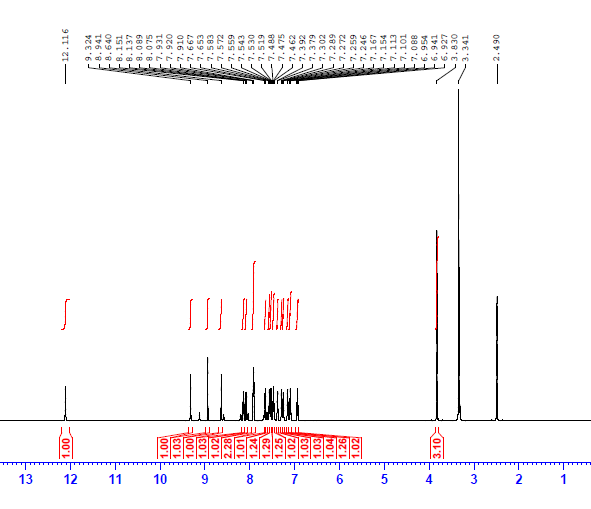


**(3o) H^1^NMR**


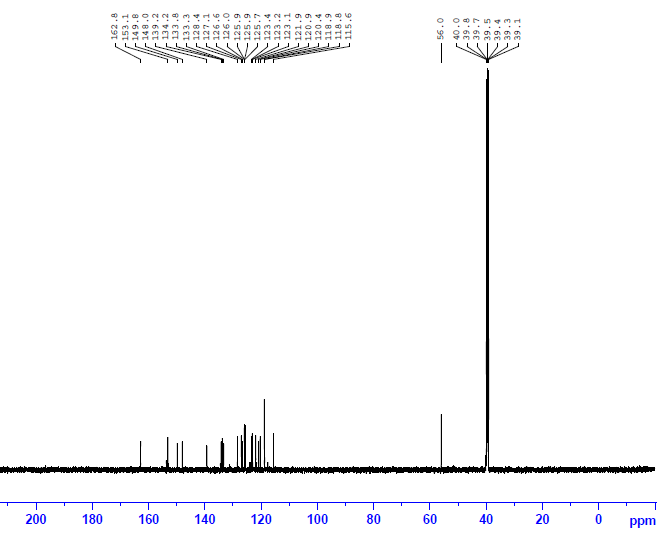


**(3o) C^13^NMR**


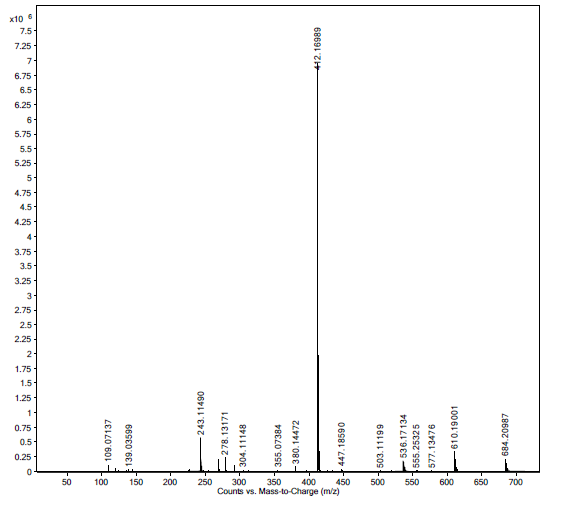


**(3o) Mass spectrum**

**(3p)** (E)-1-(2-((2-hydroxy-3-methoxybenzylidene)amino)phenyl)-3-(4-methoxyphenyl)urea

C_22_H_21_N_3_O_4_(391.43)
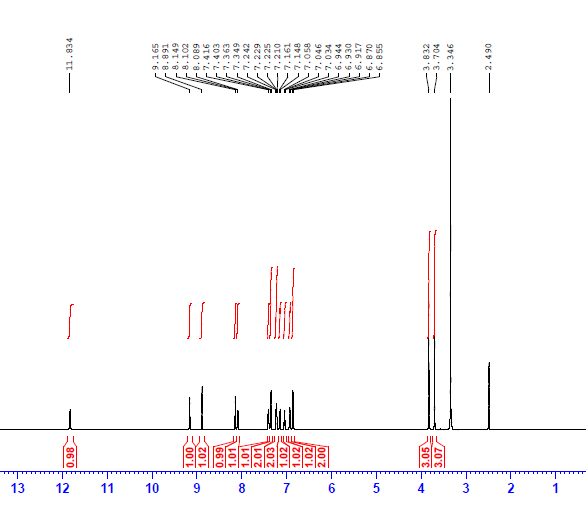


**(3p) H^1^NMR**


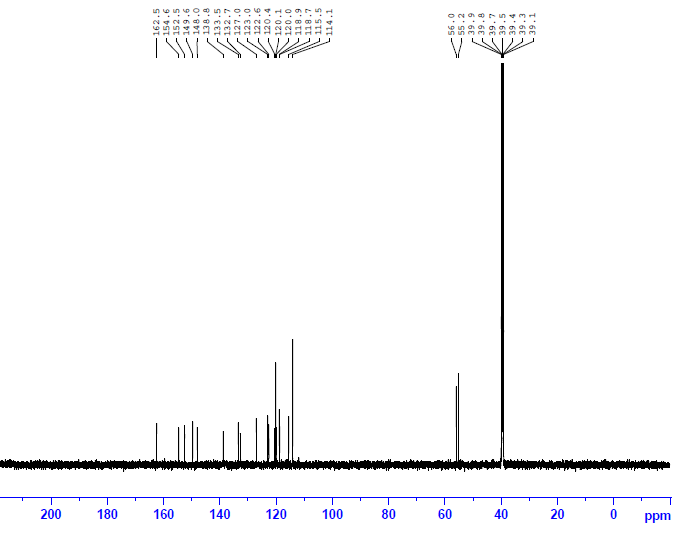


**(3p) C^13^NMR**


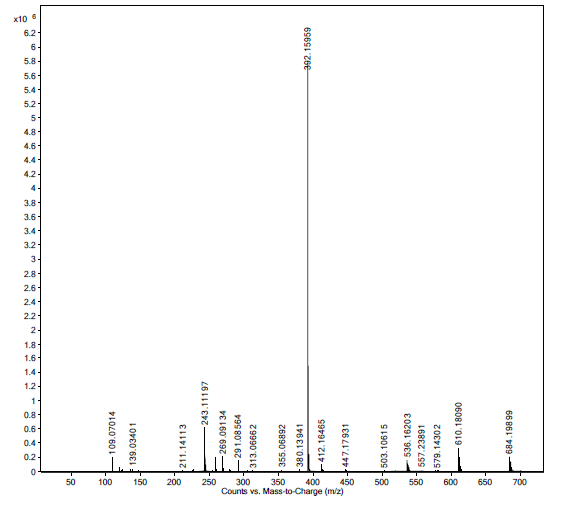


**(3p) Mass spectrum**

**(3q)** (E)-1-(4-chlorophenyl)-3-(2-((2-hydroxy-3-methoxybenzylidene)amino)phenyl)urea

C_21_H_18_ClN_3_O_3_ (395.84)
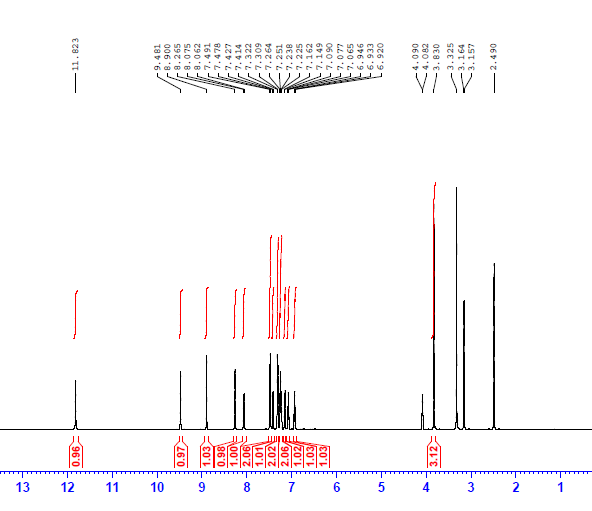


**(3q) H^13^NMR**


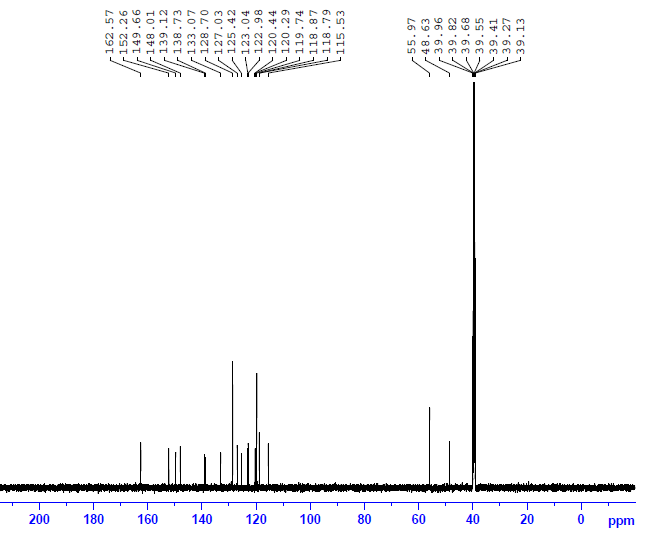


**(3q) C^13^NMR**


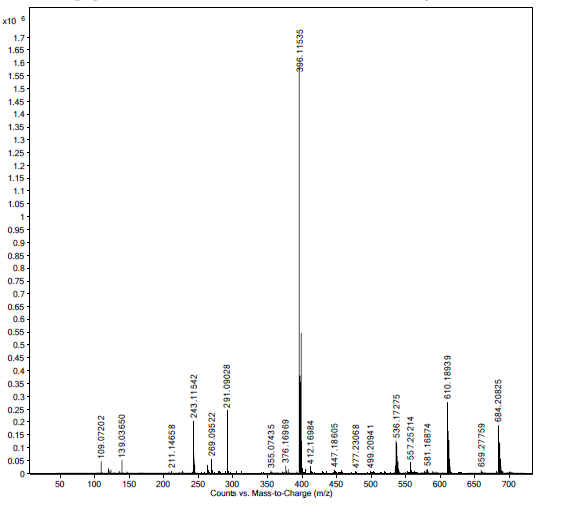


**(3q) Mass spectrum**

**(3r)** (E)-1-(2-((2-hydroxy-3-methoxybenzylidene)amino)phenyl)-3-(o-tolyl)urea

C_22_H_21_N_3_O_3_(375.43)
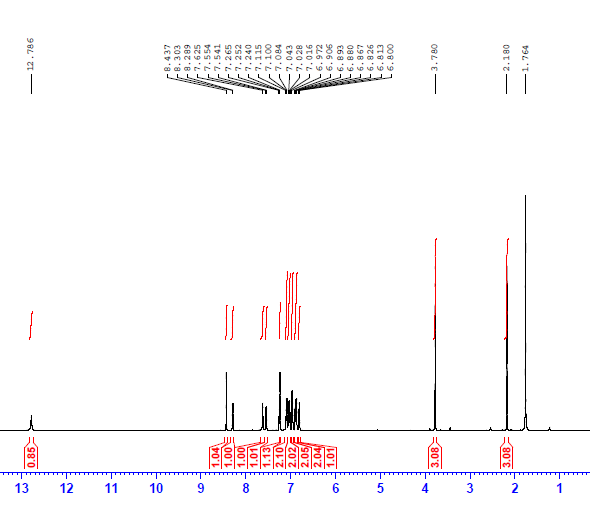


**(3r) H^1^NMR**


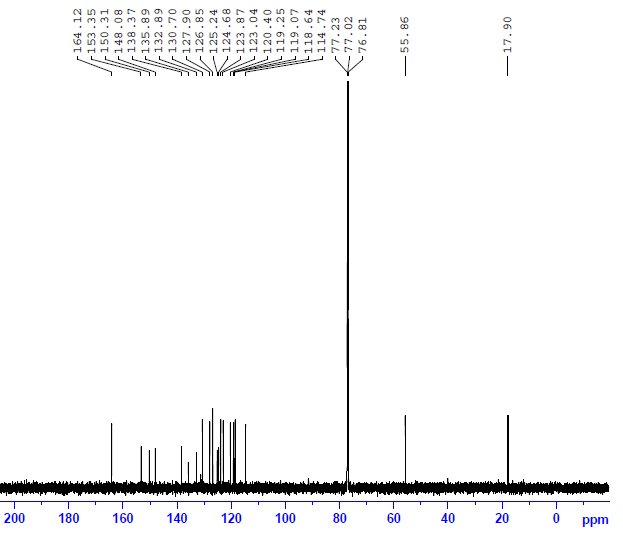


**(3r) C^13^NMR**


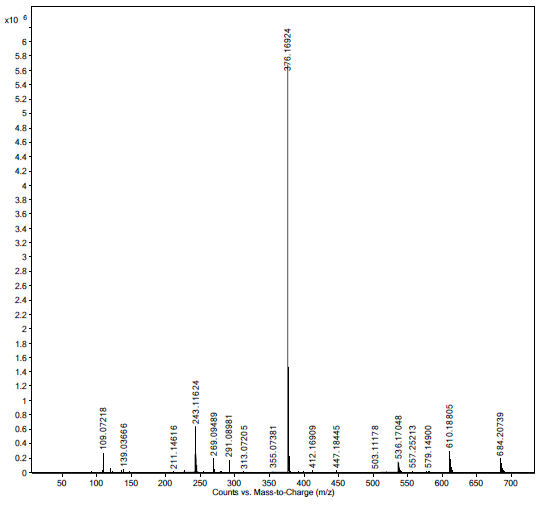


**(3r) Mass spectrum**

**(3s)** (E)-1-(2-((2-hydroxy-3-methoxybenzylidene)amino)phenyl)-3-(m-tolyl)urea

C_22_H_21_N_3_O_3_(375.43)
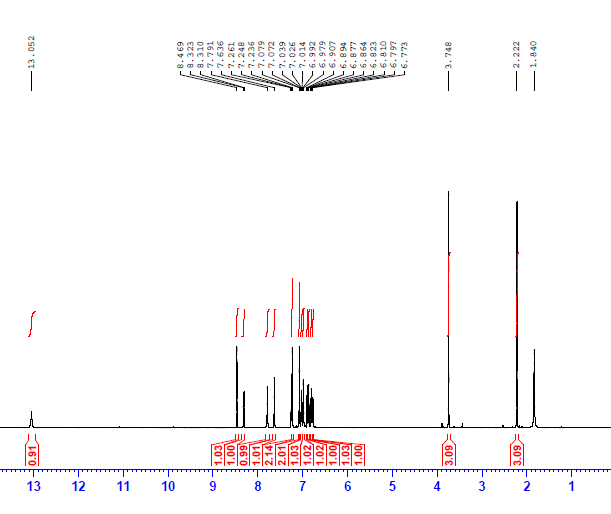


**(3s) H^1^NMR**


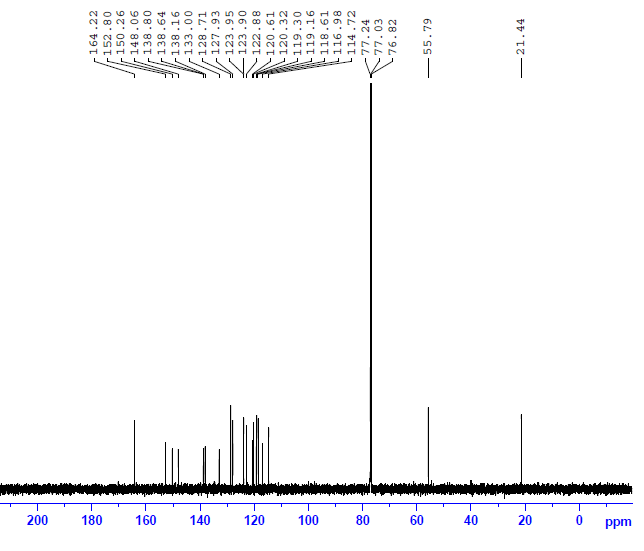


**(3s) C^13^NMR**


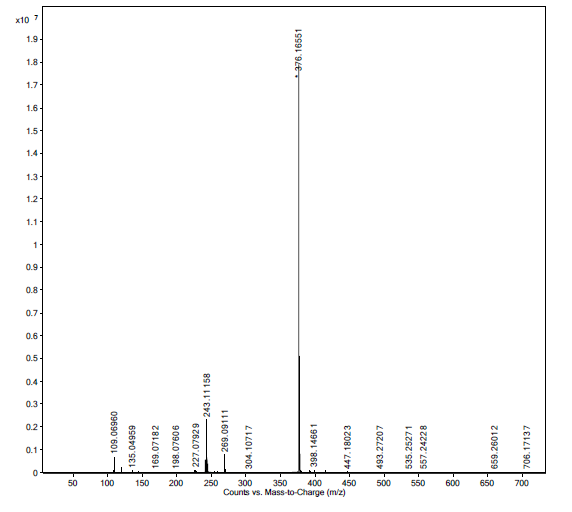


**(3s) Mass spectrum**

**(3t)** (E)-1-(2-((2-hydroxy-3-methoxybenzylidene)amino)phenyl)-3-(p-tolyl)urea

C_22_H_21_N_3_O_3_ (375.43)


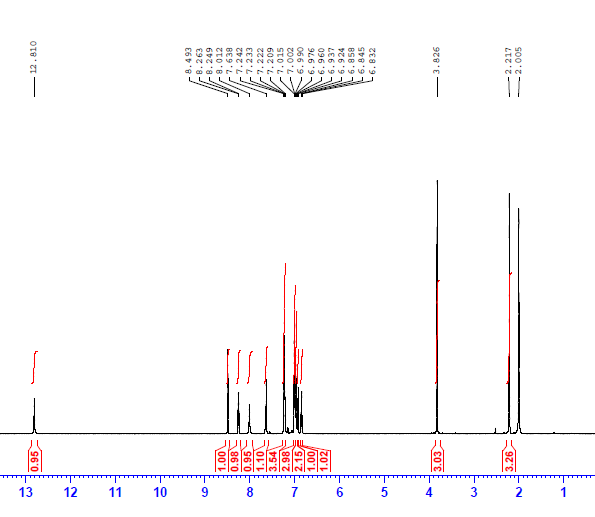


**(3t) H^13^NMR**


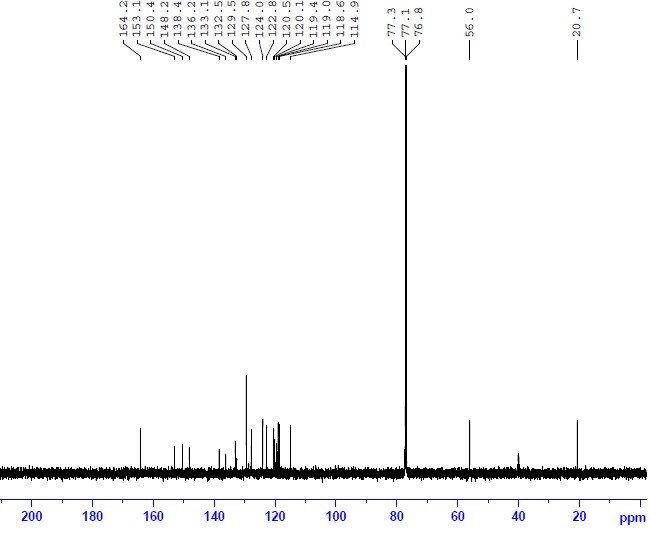
**(3t) C^13^NMR**


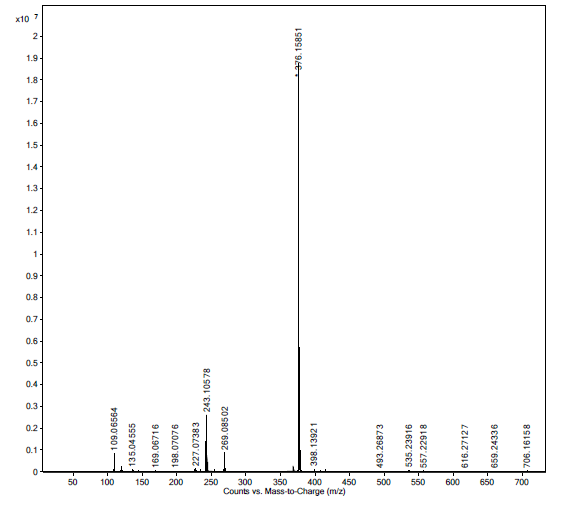


**(3t) Mass spectrum**

**(3u)** (E)-1-(2-((2-hydroxy-3-methoxybenzylidene)amino)phenyl)-3-phenylurea

C_21_H_19_N_3_O_3_ (361.40)


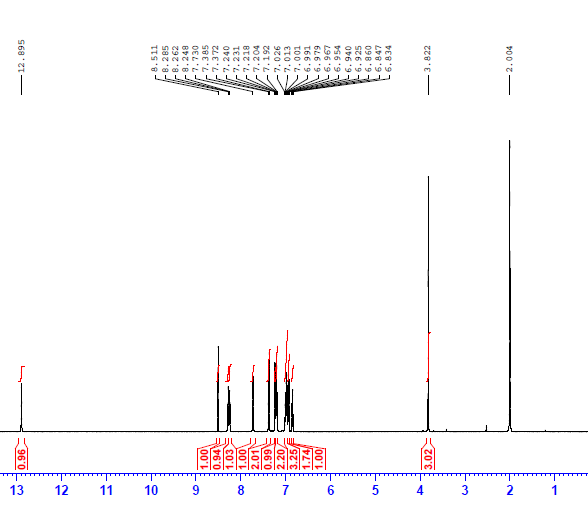


**(3u) H^13^NMR**


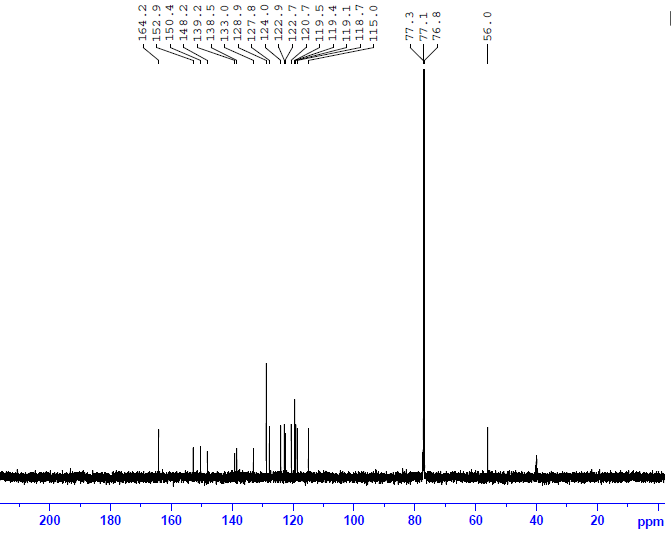


**(3u) C^13^NMR**


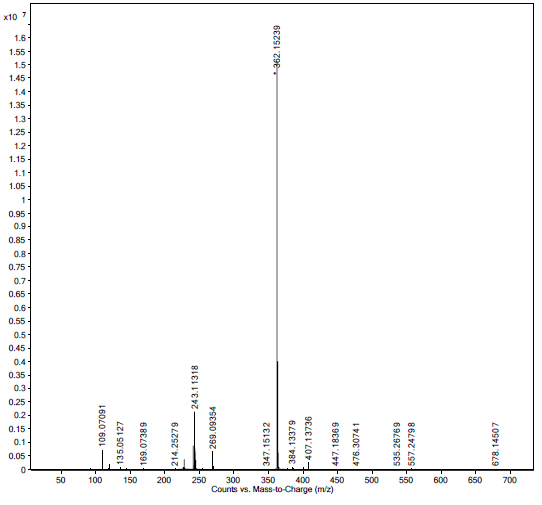


**(3u) Mass spectrum**

**(3v)** (E)-1-(4-acetylphenyl)-3-(2-((2-hydroxy-3-methoxybenzylidene)amino)phenyl)urea

C_23_H_21_N_3_O_4_(403.44)
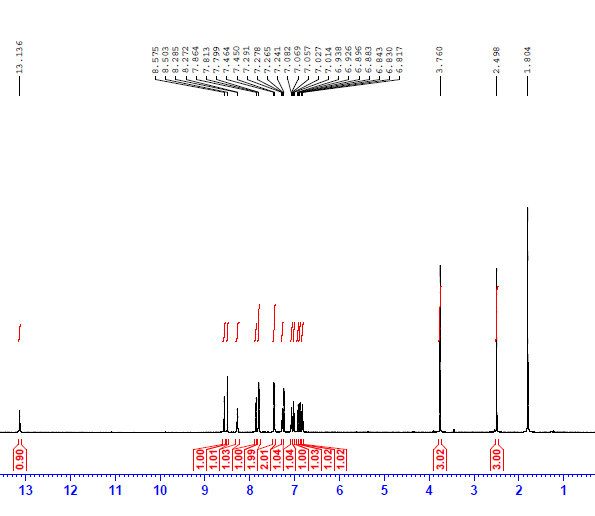


**(3v) H^1^NMR**


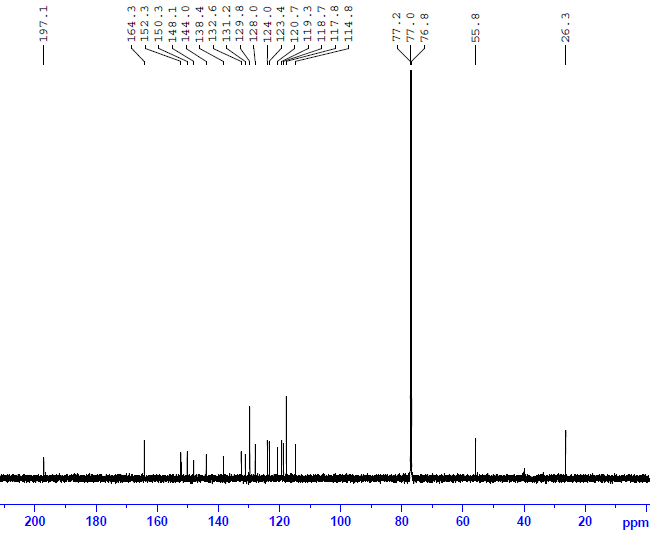


**(3v) C^13^NMR**


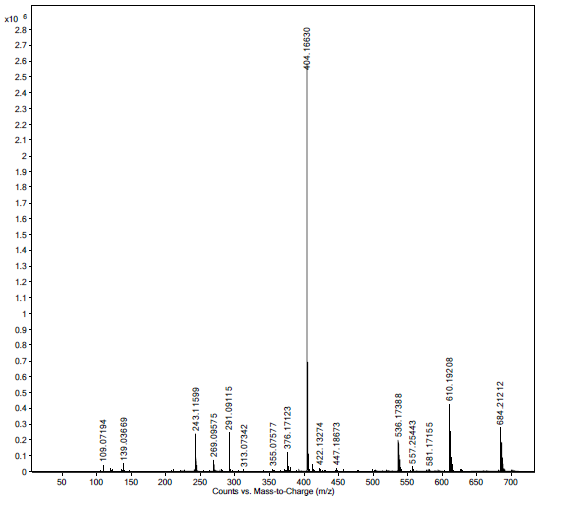


**(3v) Mass spectrum**

**(3w)** (E)-1-(4-chlorophenyl)-3-(2-((4-hydroxy-3-methoxybenzylidene)amino)phenyl)urea

C_21_H_18_ClN_3_O_3_ (395.84)


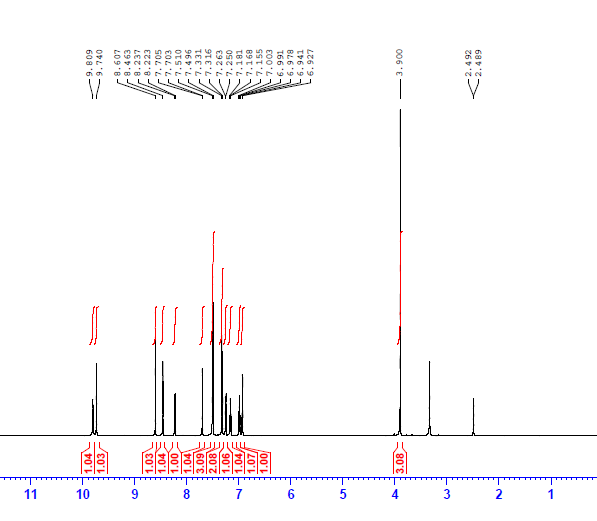


**(3w) H^1^NMR**


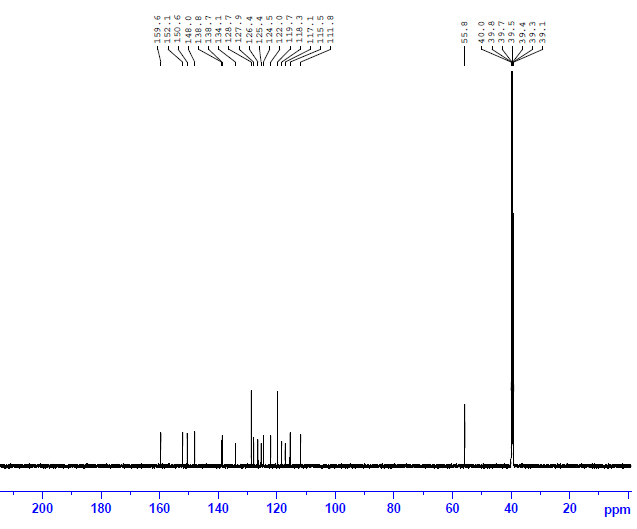


**(3w) C^13^NMR**


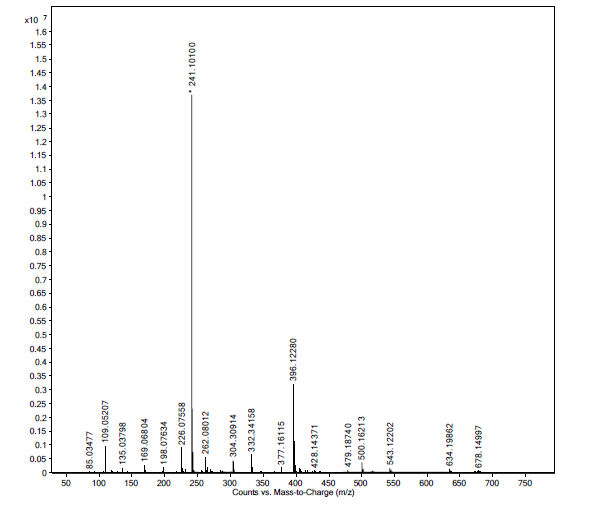


**(3w) Mass spectrum**

**(3x)** **(E)-1-(2-((4-hydroxy-3-methoxybenzylidene)amino)phenyl)-3-(o-tolyl)urea**

C_22_H_21_N_3_O_3_ (375.43)


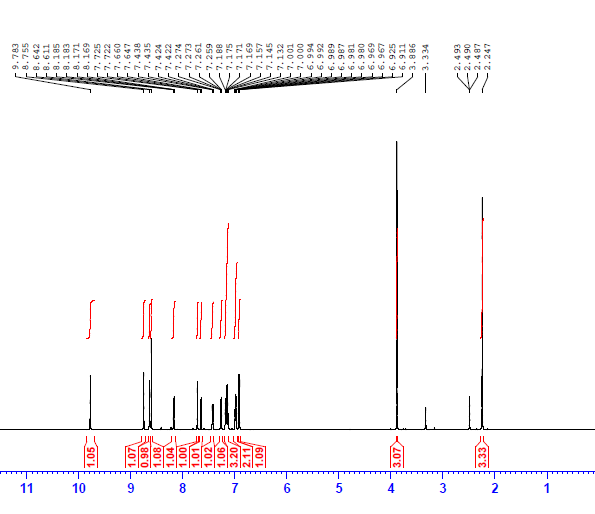


**(3x) H^13^NMR**


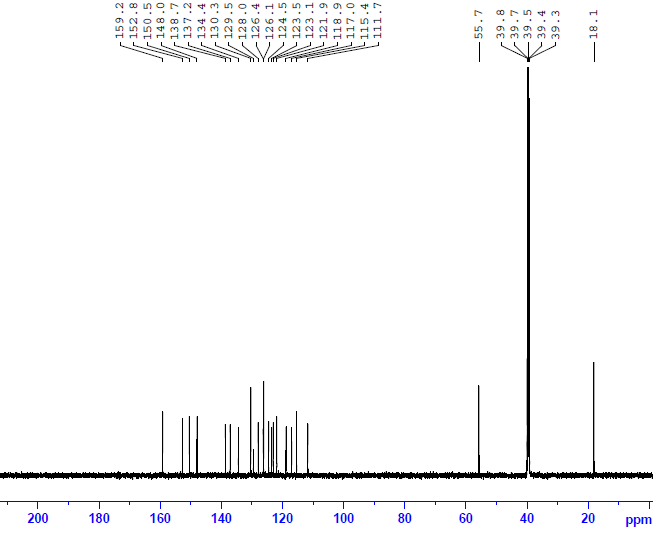


**(3x) C^13^NMR**


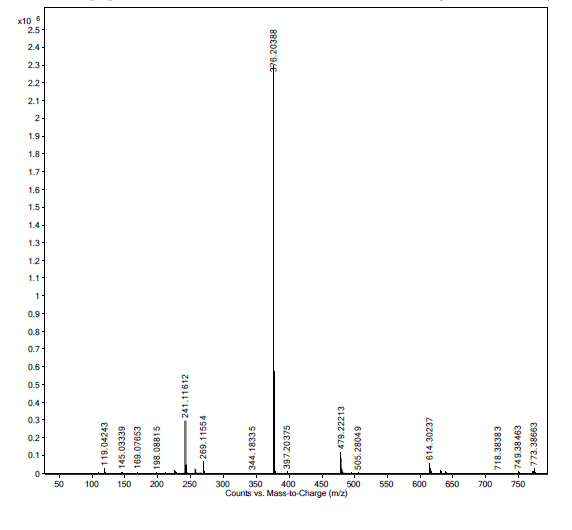


**(3x) Mass spectrum**

**(3y)** (E)-1-(2-((4-hydroxy-3-methoxybenzylidene)amino)phenyl)-3-(p-tolyl)urea

C_22_H_21_N_3_O_3_(375.43)


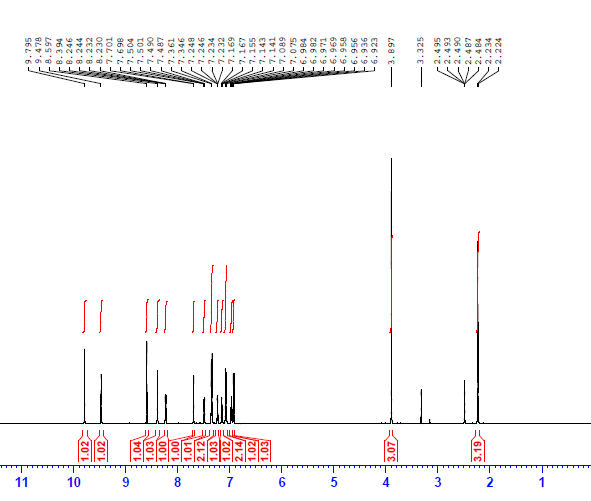


**(3y) H^1^NMR**


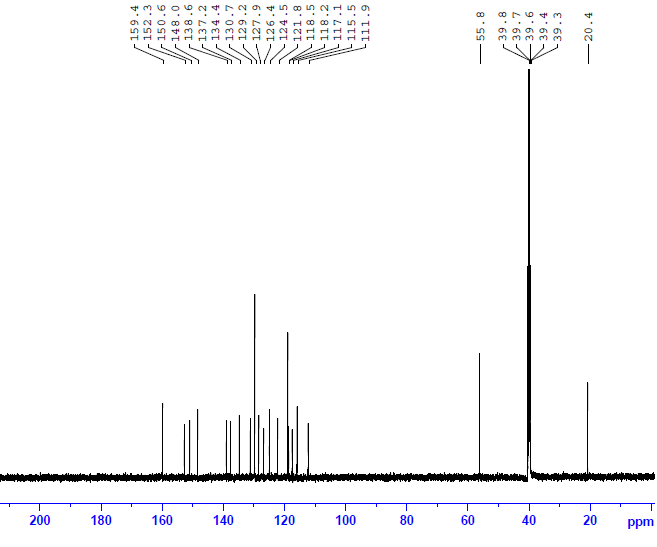


**(3y) C^13^NMR**


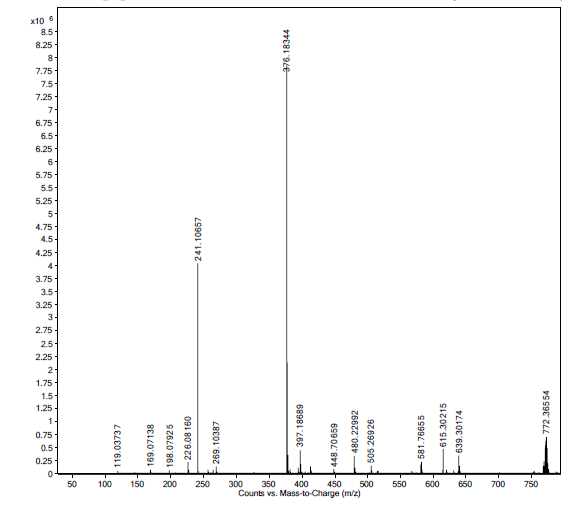


**(3y) Mass spectrum**

**Table S1. Predicted Physicochemical Properties of Compounds**

| **Molecule** | **MW** | **Fraction Csp3** | **RB** | **HBA** | **HBD** | **MR** | **TPSA (Å^2^)** | **iLOGP** | **Log S** | **Class** |
| --- | --- | --- | --- | --- | --- | --- | --- | --- | --- | --- |
| 3a | 409.87 | 0.09 | 8 | 4 | 3 | 117.19 | 82.95 | 3.62 | -5.08 | MS |
| 3b | 393.41 | 0.09 | 8 | 5 | 3 | 112.14 | 82.95 | 3.9 | -4.65 | MS |
| 3c | 393.41 | 0.09 | 8 | 5 | 3 | 112.14 | 82.95 | 3.82 | -4.65 | MS |
| 3d | 425.48 | 0.08 | 8 | 4 | 3 | 129.69 | 82.95 | 3.78 | -5.62 | MS |
| 3e | 405.45 | 0.13 | 9 | 5 | 3 | 118.67 | 92.18 | 4.09 | -4.56 | MS |
| 3f | 409.87 | 0.09 | 8 | 4 | 3 | 117.19 | 82.95 | 4.08 | -5.08 | MS |
| 3g | 389.45 | 0.13 | 8 | 4 | 3 | 117.15 | 82.95 | 3.76 | -4.79 | MS |
| 3h | 389.45 | 0.13 | 8 | 4 | 3 | 117.15 | 82.95 | 3.67 | -4.79 | MS |
| 3i | 389.45 | 0.13 | 8 | 4 | 3 | 117.15 | 82.95 | 3.95 | -4.79 | MS |
| 3j | 375.42 | 0.09 | 8 | 4 | 3 | 112.18 | 82.95 | 3.71 | -4.49 | MS |
| 3k | 417.46 | 0.12 | 9 | 5 | 3 | 122.38 | 100.02 | 3.51 | -4.44 | MS |
| 3l | 395.84 | 0.05 | 7 | 4 | 3 | 112.38 | 82.95 | 3.14 | -4.85 | MS |
| 3m | 379.38 | 0.05 | 7 | 5 | 3 | 107.33 | 82.95 | 2.99 | -4.41 | MS |
| 3n | 379.38 | 0.05 | 7 | 5 | 3 | 107.33 | 82.95 | 2.96 | -4.41 | MS |
| 3o | 411.45 | 0.04 | 7 | 4 | 3 | 124.88 | 82.95 | 3.59 | -5.38 | MS |
| 3p | 391.42 | 0.09 | 8 | 5 | 3 | 113.87 | 92.18 | 3.44 | -4.32 | MS |
| 3q | 395.84 | 0.05 | 7 | 4 | 3 | 112.38 | 82.95 | 3.02 | -4.85 | MS |
| 3r | 375.42 | 0.09 | 7 | 4 | 3 | 112.34 | 82.95 | 3.55 | -4.56 | MS |
| 3s | 375.42 | 0.09 | 7 | 4 | 3 | 112.34 | 82.95 | 3.49 | -4.56 | MS |
| 3t | 375.42 | 0.09 | 7 | 4 | 3 | 112.34 | 82.95 | 3.71 | -4.56 | MS |
| 3u | 361.39 | 0.05 | 7 | 4 | 3 | 107.37 | 82.95 | 2.82 | -4.25 | MS |
| 3v | 403.43 | 0.09 | 8 | 5 | 3 | 117.57 | 100.02 | 3.14 | -4.2 | MS |
| 3w | 395.84 | 0.05 | 7 | 4 | 3 | 112.38 | 82.95 | 3.31 | -4.85 | MS |
| 3x | 375.42 | 0.09 | 7 | 4 | 3 | 112.34 | 82.95 | 3.38 | -4.56 | MS |
| 3y | 375.42 | 0.09 | 7 | 4 | 3 | 112.34 | 82.95 | 3.68 | -4.56 | MS |

MW = Molecular Weight, RB = Number of Rotatable bonds, HBA = hydrogen bond acceptor atoms, HBD = hydrogen bond donor atoms, MR = molar refractivity, TPSA = topological polar surface area, LogP = Partition coefficient (octanol/water), Log S = water solubility, MS = Moderately Soluble

**Table S2. Predicted Pharmacokinetics of Compounds**

| **Molecule** | **GIA** | **BBBP** | **Pgp S** | **CYP1A2I** | **CYP2C19I** | **CYP2C9I** | **CYP2D6I** | **CYP3A4I** | **log Kp (cm/s)** |
| --- | --- | --- | --- | --- | --- | --- | --- | --- | --- |
| 3a | High | No | No | Yes | Yes | Yes | Yes | No | -5.68 |
| 3b | High | No | No | No | Yes | Yes | Yes | No | -5.95 |
| 3c | High | No | No | No | Yes | Yes | Yes | No | -5.95 |
| 3d | High | No | No | Yes | Yes | Yes | Yes | No | -5.33 |
| 3e | High | No | No | No | Yes | Yes | Yes | No | -6.12 |
| 3f | High | No | No | Yes | Yes | Yes | Yes | No | -5.68 |
| 3g | High | No | No | Yes | Yes | Yes | Yes | Yes | -5.74 |
| 3h | High | No | No | Yes | Yes | Yes | Yes | Yes | -5.74 |
| 3i | High | No | No | Yes | Yes | Yes | Yes | Yes | -5.74 |
| 3j | High | No | No | Yes | Yes | Yes | Yes | No | -5.91 |
| 3k | High | No | No | No | Yes | Yes | Yes | Yes | -6.4 |
| 3l | High | No | No | Yes | Yes | Yes | Yes | No | -5.85 |
| 3m | High | No | No | No | Yes | Yes | Yes | No | -6.13 |
| 3n | High | No | No | No | Yes | Yes | Yes | No | -6.13 |
| 3o | High | No | No | Yes | Yes | Yes | Yes | No | -5.51 |
| 3p | High | No | No | Yes | Yes | Yes | Yes | No | -6.29 |
| 3q | High | No | No | Yes | Yes | Yes | Yes | No | -5.85 |
| 3r | High | No | No | Yes | Yes | Yes | Yes | Yes | -5.91 |
| 3s | High | No | No | Yes | Yes | Yes | Yes | Yes | -5.91 |
| 3t | High | No | No | Yes | Yes | Yes | Yes | Yes | -5.91 |
| 3u | High | No | No | Yes | Yes | Yes | Yes | No | -6.09 |
| 3v | High | No | No | No | Yes | Yes | Yes | Yes | -6.57 |
| 3w | High | No | No | Yes | Yes | Yes | Yes | No | -5.85 |
| 3x | High | No | No | Yes | Yes | Yes | Yes | Yes | -5.91 |
| 3y | High | No | No | Yes | Yes | Yes | Yes | Yes | -5.91 |

GIA = Gastrointestinal absorption, BBBP = Blood brain barrier Permeant, Pgp S = P glycoprotein substrate, I = Inhibitor, log Kp = skin permeation

**Table S3. Predicted Druglikeness and Medicinal Chemistry of Compounds**

| **Molecule** | **Lipinski #V** | **Ghose #V** | **Veber #V** | **Egan #V** | **Muegge #V** | **BA** | **PAINS #alerts** | **Leadlikeness #V** | **SA** |
| --- | --- | --- | --- | --- | --- | --- | --- | --- | --- |
| 3a | 0 | 0 | 0 | 0 | 0 | 0.55 | 0 | 3 | 3.29 |
| 3b | 0 | 0 | 0 | 0 | 0 | 0.55 | 0 | 3 | 3.27 |
| 3c | 0 | 0 | 0 | 0 | 0 | 0.55 | 0 | 3 | 3.33 |
| 3d | 0 | 1 | 0 | 1 | 1 | 0.55 | 0 | 3 | 3.54 |
| 3e | 0 | 0 | 0 | 0 | 0 | 0.55 | 0 | 3 | 3.47 |
| 3f | 0 | 0 | 0 | 0 | 0 | 0.55 | 0 | 3 | 3.29 |
| 3g | 0 | 0 | 0 | 0 | 0 | 0.55 | 0 | 3 | 3.43 |
| 3h | 0 | 0 | 0 | 0 | 0 | 0.55 | 0 | 3 | 3.44 |
| 3i | 0 | 0 | 0 | 0 | 0 | 0.55 | 0 | 3 | 3.41 |
| 3j | 0 | 0 | 0 | 0 | 0 | 0.55 | 0 | 3 | 3.31 |
| 3k | 0 | 0 | 0 | 0 | 0 | 0.55 | 0 | 2 | 3.39 |
| 3l | 0 | 0 | 0 | 0 | 0 | 0.55 | 0 | 2 | 3.16 |
| 3m | 0 | 0 | 0 | 0 | 0 | 0.55 | 0 | 1 | 3.14 |
| 3n | 0 | 0 | 0 | 0 | 0 | 0.55 | 0 | 1 | 3.21 |
| 3o | 0 | 0 | 0 | 0 | 0 | 0.55 | 0 | 2 | 3.41 |
| 3p | 0 | 0 | 0 | 0 | 0 | 0.55 | 0 | 2 | 3.34 |
| 3q | 0 | 0 | 0 | 0 | 0 | 0.55 | 0 | 2 | 3.17 |
| 3r | 0 | 0 | 0 | 0 | 0 | 0.55 | 0 | 2 | 3.3 |
| 3s | 0 | 0 | 0 | 0 | 0 | 0.55 | 0 | 2 | 3.31 |
| 3t | 0 | 0 | 0 | 0 | 0 | 0.55 | 0 | 2 | 3.28 |
| 3u | 0 | 0 | 0 | 0 | 0 | 0.55 | 0 | 1 | 3.18 |
| 3v | 0 | 0 | 0 | 0 | 0 | 0.55 | 0 | 2 | 3.26 |
| 3w | 0 | 0 | 0 | 0 | 0 | 0.55 | 0 | 2 | 3.09 |
| 3x | 0 | 0 | 0 | 0 | 0 | 0.55 | 0 | 2 | 3.22 |
| 3y | 0 | 0 | 0 | 0 | 0 | 0.55 | 0 | 2 | 3.19 |

#V = Number of violations, BA = Bioavailability Score, SA = Synthetic Accessibility,
